# LAST-seq: single-cell RNA sequencing by direct amplification of single-stranded RNA without prior reverse transcription and second-strand synthesis

**DOI:** 10.1186/s13059-023-03025-5

**Published:** 2023-08-09

**Authors:** Jun Lyu, Chongyi Chen

**Affiliations:** Laboratory of Biochemistry and Molecular Biology, National Cancer Institute, National Institutes of Health, Bethesda, MD 20892 USA

## Abstract

Existing single-cell RNA sequencing (scRNA-seq) methods rely on reverse transcription (RT) and second-strand synthesis (SSS) to convert single-stranded RNA into double-stranded DNA prior to amplification, with the limited RT/SSS efficiency compromising RNA detectability. Here, we develop a new scRNA-seq method, Linearly Amplified Single-stranded-RNA-derived Transcriptome sequencing (LAST-seq), which directly amplifies the original single-stranded RNA molecules without prior RT/SSS. LAST-seq offers a high single-molecule capture efficiency and a low level of technical noise for single-cell transcriptome analyses. Using LAST-seq, we characterize transcriptional bursting kinetics in human cells, revealing a role of topologically associating domains in transcription regulation.

**Supplementary Information:**

The online version contains supplementary material available at 10.1186/s13059-023-03025-5.

## Background

Single-cell transcriptome analyses have been driven by the invention of scRNA-seq methods [1–5]. Recent technical advances have focused on improving the performance of digital counting by unique molecular identifiers (UMIs) [6–8], enhancing the cellular throughput while lowering the cost [9–17], optimizing individual steps in the protocol [8, 18, 19], and miniaturization [19–21]. The underlying chemistry, however, remains unchanged and depends on the same fundamental step of RT/SSS prior to the amplification of single-stranded RNA (ssRNA) molecules. While RT remains reliant on reverse transcriptase, current scRNA-seq assays employ various SSS strategies with a limited efficiency, including the use of terminal transferase [1] or template switching [2, 3, 6, 8] to create cDNA priming sites for PCR, employing RNase H and DNA Pol to convert the RNA/cDNA hybrid to double-stranded DNA [4, 7] for in vitro transcription, random annealing to the single-stranded cDNA for extension [22], or Tn5 tagmentation of the RNA/cDNA hybrid [23].

Regardless of the specific protocol, the inevitable RT/SSS with a limited efficiency in existing scRNA-seq methods ultimately compromises the single-molecule capture efficiency of the original RNA molecules. Low RNA capture efficiency can lead to measurement inaccuracies (Additional file 1: Fig. S1a), undermining the ability to distinguish cell types with a subtle difference in the gene expression level. Low RNA capture efficiency also adds a high level of technical noise in the single-cell transcriptome data (Additional file 1: Fig. S1b), hindering accurate characterization of cell-to-cell variation and gene expression noise.

To address this limitation, we developed a new scRNA-seq assay called LAST-seq. Rather than relying on the inefficient RT/SSS prior to RNA amplification, LAST-seq directly amplifies the original ssRNA molecules in single cells in a linear fashion, achieving a high single-molecule capture efficiency and a low level of technical noise compared to existing scRNA-seq methods. Using LAST-seq, we characterized gene expression noise and transcriptional bursting kinetics, and investigated the regulation of transcriptional activities by topologically associating domains (TADs) in human cells.

## Results

### *Efficient T7 *in vitro* transcription of single-stranded RNA templates*

T7 in vitro transcription (IVT) of double-stranded DNA (dsDNA) templates to generate antisense RNA (aRNA) [24] has been widely used to achieve linear amplification in single-cell assays [4, 7, 25]. Here, we tested the efficiency of T7 IVT with a 20-nt ssRNA template as compared to a dsDNA template of the same sequence. When an 18-bp dsDNA linker was included between the upstream T7 promoter and the downstream ssRNA template, we found that T7 IVT was also efficient (Additional file 1: Fig. S2a), consistent with a previous report that used a 14-nt single-stranded template [26]. Moreover, T7 IVT reactions showed equal efficiency regardless of the presence of various modifications and structures at the junction site between the dsDNA linker and the ssRNA template (Additional file 1: Fig. S2a). Importantly, the length of aRNA generated during the IVT of ssRNA templates could exceed the 20-nt short distance, reaching up to 200–500 nt before the final falloff of the elongating polymerase from the single-stranded template (Additional file 1: Fig. S2b). For the tested ssRNA templates, we observed more than 500-fold linear amplification ~ 250-nt downstream of the T7 promoter and close to zero-fold ~ 1000-nt downstream of the promoter (Additional file 1: Fig. S2c). The different amplicon lengths and amplification fold between the tested ssRNA templates were likely due to their distinct template sequences, as the same phenomenon was reported for the canonical T7 IVT of double-stranded DNA templates [27].

### Direct linear amplification of single-cell RNA molecules in LAST-seq enables a high detection sensitivity

**Fig. 1:**
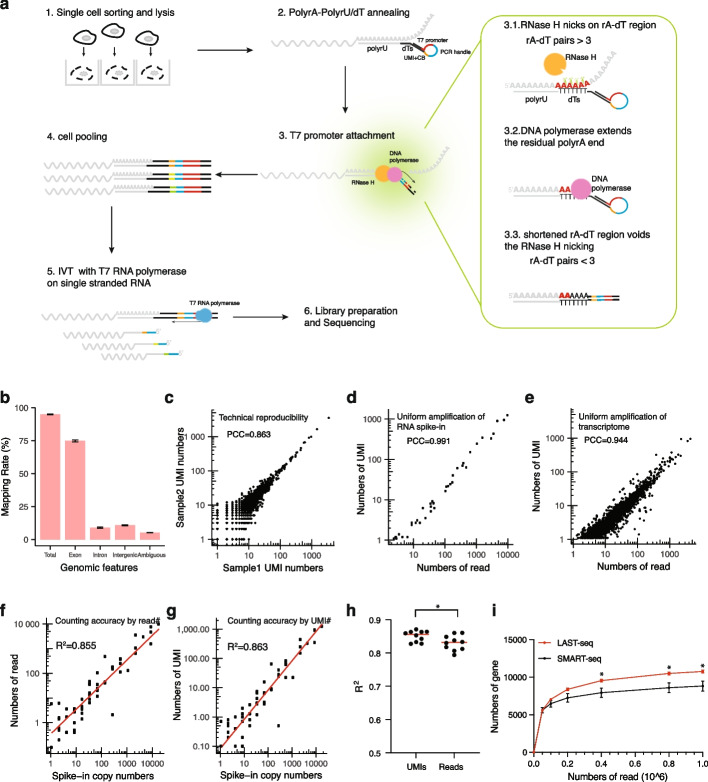
Workflow and performance of LAST-seq. **a** Schematic showing each step of the LAST-seq protocol. **b** Mapping rates of the LAST-seq reads to different genomic regions. The standard error of the mean (SEM) error bar is calculated from 10 cells. **c** Technical reproducibility of the LAST-seq data determined by the Pearson’s correlation coefficient (PCC) between two replicates using 15-pg HEK293T-extracted total RNA as the input. **d** Correlation between the number of UMIs and sequencing reads in the LAST-seq data of the ERCC RNA spike-in. **e** Correlation between the number of UMIs and the sequencing reads in the LAST-seq data of single HEK293T cells. **f** Linear correlation between the number of sequencing reads in the LAST-seq data and the input copy numbers of the ERCC RNA spike-in. **g** Linear correlation between the number of UMIs in the LAST-seq data and the input copy numbers of the ERCC RNA spike-in. **h** Coefficient of determination (*R*^2^) of the linear regression in (**f**) and (**g**), from 10 samples. The statistical analysis was performed by Welch *t*-test (*p ≤ 0.05). **i** Number of detected genes in single HEK293T cells with various sequencing depths. The SEM error bar is calculated from 10 and 9 cells for LAST-seq and SMART-seq, respectively

Based on the observation that T7 IVT can directly amplify ssRNA templates, generating sufficiently long aRNA molecules with a reasonable linear amplification fold, we developed the LAST-seq assay as an RT/SSS-free method for single-cell RNA amplification and sequencing (Fig. 1a). First, the poly-A tail of each mRNA molecule in the single-cell lysate is annealed to the rU-dT overhang of a specially made LAST-seq primer (Additional file 1: Fig. S3). Next, the short patch of rA/dT hybrid is nicked and extended to attach a T7 promoter to the 3′-end of each mRNA molecule, which remains unchanged and in the original single-stranded status. Finally, T7 IVT of the ssRNA templates is performed to directly and linearly amplify each original single-cell mRNA molecule into hundreds of aRNA copies, followed by single-cell transcriptome library preparation based on the bulk-level aRNA products.

We applied the LAST-seq assay to probe the transcriptome of single HEK293T cells. The LAST-seq assay showed a reasonable mapping rate (Fig. 1b), a high technical reproducibility (Fig. 1c and Additional file 1: S4a-b), and an expected 3′-end enrichment of the sequencing read coverage (Additional file 1: Fig. S4c), thus not suitable for full-length scRNA-seq studies. Notably, using the ERCC RNA spike-in mix, no significant amplification bias was detected between different RNA spike-in species (Fig. 1d), suggesting a generally uniform IVT linear amplification fold of ssRNA templates in the LAST-seq assay, regardless of the template sequence. Targeting the GAPDH transcript, we estimated the linear amplification in the LAST-seq assay to be a few 100-fold, in agreement with results from the arbitrary ssRNA templates (Additional file 1: Fig. S4d). Moreover, as predicted for the linear amplification, strong correlations were observed between the number of UMIs and the number of sequencing reads for transcriptomic RNAs (Fig. 1e), with the former showed a smaller variation than the later between technical replicates, demonstrating the benefit of counting UMIs to further remove amplification bias (Additional file 1: Fig. S4e). Not surprisingly, the input RNA spike-in copy number correlated well with the number of corresponding sequencing reads or UMIs (Fig. 1f, g), and the number of UMIs was slightly better than the number of sequencing reads to reflect the input copy number (Fig. 1h).

We then compared the performance of LAST-seq assay to existing scRNA-seq methods, particularly the widely used SMART-seq assay, using the commercially available SMART-seqV4 kit (Additional file 1: Fig. S4f). After performing LAST-seq and SMART-seq on flow-sorted single HEK293T cells, we observed a reasonable correlation between the single-cell transcriptome generated by the two methods (Additional file 1: Fig. S4g), suggesting that LAST-seq can recapitulate the results of SMART-seq. Next, we reasoned that elimination of inefficient RT/SSS steps by direct amplification of the original RNA molecules in LAST-seq could lead to a high detection sensitivity. Indeed, LAST-seq showed a better gene detectability than SMART-seq (Fig. 1i). The conclusion was further supported by the RNA spike-in experiment, where LAST-seq also demonstrated a higher detection rate of the RNA spike-in, given the same input copy number of each spike-in species (Additional file 1: Fig. S4h-i).

### LAST-seq offers a high single-molecule RNA capture efficiency

Another scRNA-seq method, CEL-seq [4], offers a higher detection sensitivity than SMART-seq in certain applications [28]. Similar to LAST-seq, CEL-seq also employs a linear amplification scheme, but CEL-seq’s linear amplification is performed on double-stranded DNA templates after RT/SSS of the original ssRNA. Here, in addition to SMART-seq, we also evaluated the performance of LAST-seq side-by-side with CEL-seq, using the improved CEL-seq2 protocol [7]. Since both methods utilize UMIs for the digital counting of detected RNA molecules, their single-molecule capture efficiencies can be quantified for a comparison.

**Fig. 2:**
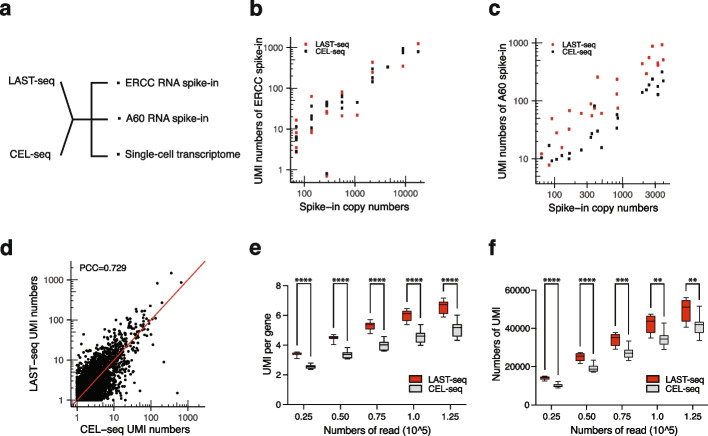
Comparison between LAST-seq and CEL-seq. **a** Experimental schematic to compare LAST-seq and CEL-seq in detecting RNA spike-in species and transcriptomic RNA molecules. **b** Capture efficiency of LAST-seq and CEL-seq using ERCC RNA spike-in as the input, with dots representing the average number of detected UMIs from 10 and 5 replicates for LAST-seq and CEL-seq, respectively. **c** Capture efficiency of LAST-seq and CEL-seq using A60 RNA spike-in as the input, with dots representing the average number of detected UMIs from 10 and 5 replicates for LAST-seq and CEL-seq, respectively. **d** Correlation of RNA level quantified by UMI numbers between LAST-seq and CEL-seq, both averaged from 10 single cells. Each dot corresponds to one gene. **e** Number of UMIs per gene comparison under various sequencing depths between LAST-seq and CEL-seq, both averaged from 10 single cells. **f** Number of total UMI per cell comparison under various sequencing depths between LAST-seq and CEL-seq, both averaged from 10 single cells. The boxplot shows the median (center line), the 25/75 percentile (bounds), and the minimum/maximum (whiskers). The statistical analysis was performed by the Welch *t*-test (**p ≤ 0.01, ***p ≤ 0.001, ****p ≤ 0.0001)

First, we carried out RNA spike-in experiments as the gold standard to quantify the single-molecule capture efficiency of scRNA-seq assays, by spiking various RNA species in the single cell to mimic the transcriptomic RNA molecules but with known input copy numbers (Fig. 2a). The single-molecule capture efficiency is reflected by the ratio between the detected copy number and the input copy number of each RNA spike-in species. In addition to the commonly used ERCC RNA spike-in mix [29], which contains a ~ 24-nt poly-A tail on the 3′ end of each spike-in species, much shorter than the poly-A tail of typical mRNA molecules in human cells [30] (Additional file 1: Fig. S5a), we also synthesized an alternative RNA spike-in mix (A60), which contains a 60-nt 3′-end poly-A tail for each RNA spike-in species to mimic human mRNA molecules (Additional file 1: Fig. S5b). Estimated from both types of RNA spike-in mix, LAST-seq offered a similar or higher single-molecule RNA capture efficiency compared to CEL-seq (Fig. 2b, c). However, the difference between different RNA spike-ins also indicated that single-molecule capture efficiency quantified by RNA spike-in experiments may be biased by the poly-A tail length [31] of the spike-in species. Specifically, the higher capture efficiency of LAST-seq for A60 RNA spike-in mix was likely due to enhanced annealing between the LAST-seq primer and the long poly-A tail, stabilizing the rA/dT hybrid for more efficient RNase H nicking and T7 promoter attachment.

Next, in order to evaluate the performance of both assays in capturing real transcriptomic RNA molecules in single human cells, we performed LAST-seq and CEL-seq on flow-sorted single HEK293T cells. First, the number of UMIs reported by both assays showed a reasonable correlation (Fig. 2d and Additional file 1: Fig. S5c), suggesting both were able to reflect the general single-cell transcriptome. After further quantification of the number of UMIs, we found LAST-seq can detect more UMIs per gene than CEL-seq for the detected genes (Fig. 2e and Additional file 1: Fig. S5d), as well as more total UMIs per cell (Fig. 2f and Additional file 1: Fig.S5e), indicating a high single-molecule capture efficiency of the original RNA molecules in single cells.

It is worth to note that despite LAST-seq can capture more copies of mRNA molecules per gene in single cells, the total number of genes LAST-seq can detect was comparable to CEL-seq (Additional file 1: Fig. S5f). We speculate that a small portion of genes may not be readily amplified by LAST-seq due to difficulties upon IVT of ssRNA templates with very few copies (Additional file 1: Fig. S5g), significant secondary structure, and/or a short poly-A tail, compromising the total number of genes it can detect. On the other hand, compared to LAST-seq and SMART-seq, CEL-seq seems to be more likely to amplify and detect other types of cellular RNA in addition to the mature mRNA (Additional file 1: Fig. S5h), resulting in an elevated level of intronic and intergenic reads (Additional file 1: Fig. S5i), which was also observed in previous studies [28, 32], and more prone to nonspecific genomic DNA amplification upon single-cell RNA amplification and sequencing (Additional file 2: Table S1), that will also contribute to the total number of genes it can apparently detect.

Finally, we concluded that LAST-seq has a higher efficiency than CEL-seq to capture the original mRNA molecules in single cells, while the two linear-amplification-based scRNA-seq methods seem to be comparable regarding the total number of genes they can detect.

### Characterization of transcriptional bursting kinetics in human cells by LAST-seq

**Fig. 3:**
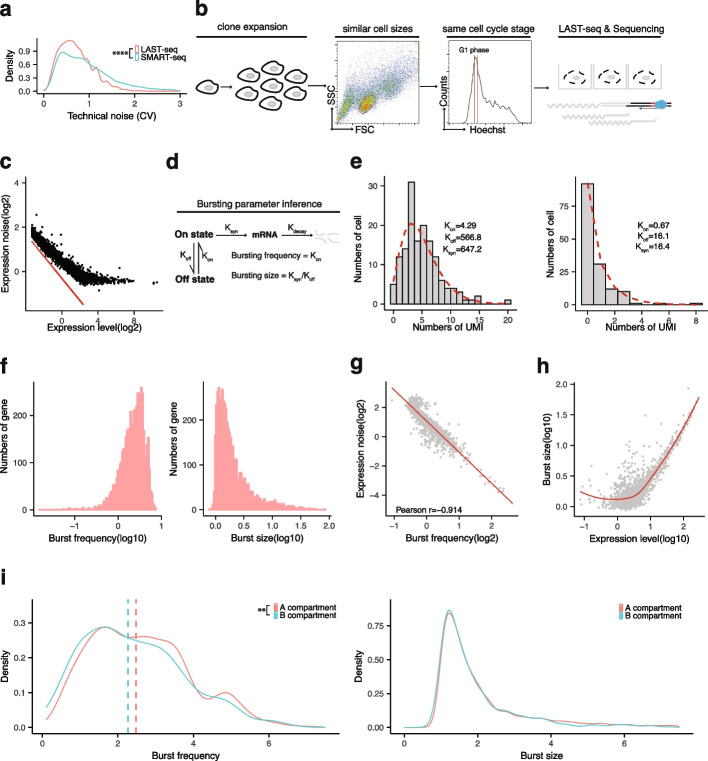
Characterization of transcriptional bursting kinetics in human cells by LAST-seq. **a** Technical noise plotted by the distribution of coefficient of variation (CV) of each mRNA species from 10 samples (LAST-seq) or 16 samples (SMART-seq). **b** Workflow of single-cell transcriptome analyses by LAST-seq to minimize the extrinsic noise. **c** Gene expression noise profile of haploid human cells. The red line represents the theoretical level of technical noise. **d** Two-state model of transcriptional bursting. **e** Examples of curve fitting to derive transcriptional burst parameters from the intrinsic noise of gene expression. **f** Transcriptome-wide distribution of burst frequency and burst size for human genes. **g** Correlation between the expression noise and burst frequency. **h** Correlation between the expression level and burst size. **i** Distribution of burst frequency and burst size for human genes located in A (*n* = 3465) and B (*n* = 645) compartments. The statistical test of (a) and (i) was performed by two-sided Kolmogorov–Smirnov test (**p ≤ 0.01, ****p ≤ 0.0001)

Thanks to its high RNA capture efficiency, LAST-seq offered a lower level of technical noise and more accurate transcriptome-wide profiling of the cell-to-cell variation than the commonly used SMART-seq (Fig. 3a), enabling a better performance in the quantitative study of gene expression noise. Here, we used LAST-seq to characterize cell-to-cell variation in human cells, quantify gene expression noise of individual genes, and derive transcriptional bursting kinetics for further investigation in the context of 3D chromatin organization.

First, we designed experiments to quantify gene expression noise from the observed cell-to-cell variation, which consists of three components, technical noise of the scRNA-seq method, extrinsic noise from various types of heterogeneity among the cell population, and intrinsic noise mainly from transcriptional bursting (Additional file 1: Fig. S6a). By using LAST-seq instead of previous scRNA-seq assays, we lowered the level of technical noise. By isolating individual cells from a single-cell clonal expansion population sharing the same genetic and epigenetic profile, and flow-sorting single G1-phase human cells gated for a narrow range of cell size and DNA content (Fig. 3b), we minimized the extrinsic noise within a homogenous cell population in terms of genetic/epigenetic background, cell size, and the cell cycle stage. Consequently, the remaining cell-to-cell variation experimentally observed was primarily due to the intrinsic noise (Fig. 3c), driven mainly by transcriptional bursting dynamics with only a minor contribution from RNA degradation kinetics [33].

Next, based on the two-state model of transcriptional bursting (Fig. 3d), we used the Poisson-Beta distribution curve fitting to derive the bursting parameters [33] of individual genes from the corresponding intrinsic noise profile (Fig. 3e). We filtered out genes deviating from the curve fitting (Additional file 1: Fig. S6g), reasoning such genes either have a high level of technical noise, or have transcription dynamics that cannot be approximated by the two-state model [34, 35], thus unsuitable for downstream study of bursting kinetics. Moreover, since the bursting activities of two alleles are often independent and unsynchronized [36], a haploid human cell line (eHAP) [37] was used in this study to avoid the need of separating allelic transcripts.

Finally, we characterized the transcriptional bursting parameters in human cells, including burst frequency and burst size (Fig. 3f). In addition to the expected *K*_off_ > *K*_on_ for most genes (Additional file 1: Fig. S6b), we observed a strong negative correlation between expression noise and burst frequency (Fig. 3g), but not burst size (Additional file 1: Fig. S6c-e). We also found a positive correlation between expression level and burst size (Fig. 3h), but not burst frequency (Additional file 1: Fig. S6f), for highly expressed genes. Oppositely, the expression level of lowly expressed genes was more correlated to burst frequency rather than burst size (Fig. 3h and Additional file 1: Fig. S6f).

Moreover, we analyzed bursting parameters for genes located in A and B compartments, which are physically separated in the 3D chromatin organization of human cells [38]. We found that genes located in the A compartment showed a slightly higher overall burst frequency, but not a larger burst size, compared to genes located in the relatively inactive B compartment (Fig. 3i), in agreement with a higher expression noise in more repressed chromatin previously observed by single-molecule FISH in a few genes [39].

### Role of topologically associating domains (TADs) in burst frequency modulation and temporal coordination of transcription activities

Hi-C analysis of human 3D chromatin organization showed the widespread existence of topologically associating domains (TADs) as self-interacting genomic regions [38]. Despite their evolutionary conservation across species and cell types that indicates a functional relevance [40, 41], the function of TADs remains to be fully defined [40, 42–48]. Although no major changes in gene expression level were observed upon genome-wide TAD disruption and rearrangement [43–46], some specific TADs have been demonstrated to regulate gene expression [40, 47, 48]. Here, we hypothesized that, instead of regulating gene expression level, TADs might modulate transcriptional burst frequency and thus influence gene expression noise and cell-to-cell variation upon cell fate determination [49]. This hypothesis was based on the strengthened and more frequent enhancer-promoter interactions (EPI) between elements within the same TADs [50] and the observation that enhancers mostly modulate burst frequency instead of burst size [33, 51, 52].

**Fig. 4:**
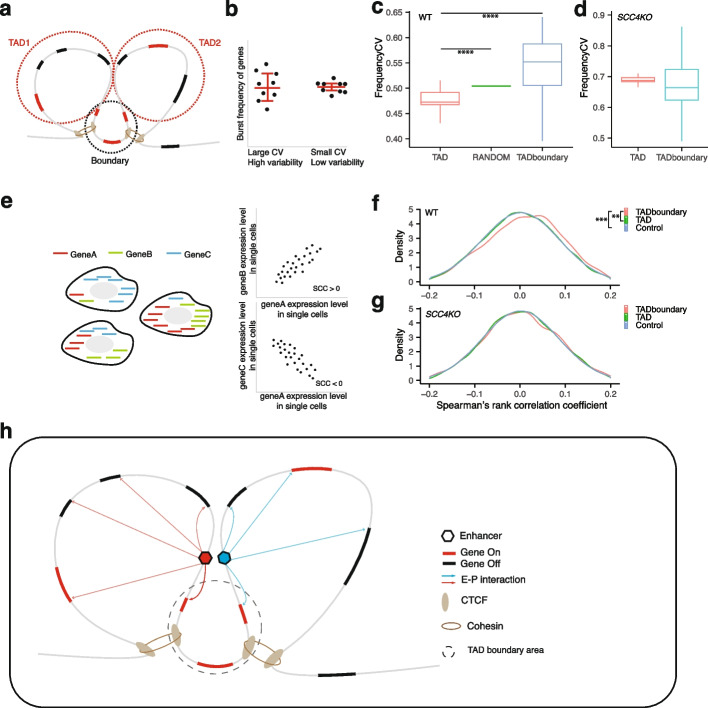
Role of TADs in burst frequency modulation and temporal correlation of transcription activities. **a** Schematic showing genes located within TADs and across TAD boundaries. **b** Burst frequency variations in groups of genes. Each dot represents the burst frequency of one gene, and the coefficient of variation (CV) of the burst frequencies is calculated as the ratio of the standard deviation to the mean. **c**, **d** Burst frequency variation in groups of genes located in the same TADs, across TAD boundaries, and randomly picked from the transcriptome (Random). The boxplot shows the median (center line), the 25/75 percentile (bounds), and farthest points within 1.5 times the interquartile range (whiskers). The statistical test was performed by two-sided Wilcoxon test (****p ≤ 0.0001). **e** Examples of positive and negative correlation of RNA level in single cells by calculating the Spearman’s rank correlation coefficient (SCC). **f**, **g** Distribution of SCC values for gene pairs located in the same TADs, across TAD boundaries, and randomly picked from the transcriptome (Control). The statistical test was performed by two-sided Kolmogorov–Smirnov test (**p ≤ 0.01, ***p ≤ 0.001). **h** Model showing burst frequency modulation within TADs and temporal correlation of transcription in the boundary

To test this hypothesis, we analyzed transcriptome-wide burst frequencies derived from our LAST-seq data. Since the TAD-related EPI is just one factor among many other TAD-independent factors affecting gene expression [53] and burst frequency [54], the burst frequency of individual genes will not demonstrate a striking TAD relevance. Consequently, burst frequencies of many genes must be analyzed together to reveal the potential role of TADs with enough statistical power. Indeed, for genes located within the same TADs (Fig. 4a), we calculated their variability in burst frequencies (Fig. 4b) and found a smaller burst frequency variation than the control group of randomly picked genes (Fig. 4c). The smaller burst frequency variation cannot be explained by *in cis* proximity of the genes located within the same TADs, since genes located across the same TAD boundaries, which are also proximal *in cis*, showed a much larger burst frequency variation (Fig. 4c). To further confirm the TAD dependence of the observed difference in burst frequency variations, we knocked out the cohesin loader complex subunit *SCC4* (Additional file 1: Fig. S7a), globally weakening and disrupting TADs in the still viable human haploid cells [55] (Additional file 1: Fig. S7b). Similar to reports in other cell types [44, 45], ~ 10% of the genes showed significant changes (> twofold) in the expression level upon cohesin loss and TAD disruption (Additional file 1: Fig. S7c). As predicted by our hypothesis, TAD disruption eliminated the burst frequency variation difference between genes located within the same TADs and genes across TAD boundaries (Fig. 4d). We did not use the degron system to disrupt TADs, since EPIs were found to be insensitive to such acute short-term depletion of cohesin or CTCF [56].

We then asked whether TADs, in addition to modulating the burst frequency, also temporally coordinate the transcription activities of genes located within them. We measured the temporal correlation of transcription activities between a pair of genes by calculating the Spearman’s rank correlation coefficient (SCC) between the gene pair’s mRNA transcript level in single cells, with a positive SCC suggesting a positive temporal correlation (Fig. 4e). From the single-cell transcriptome data generated by LAST-seq, we found no difference in the SCC distribution between gene pairs located within the same TADs and randomly picked gene pairs (Fig. 4f), suggesting a lack of temporal coordination of the transcription activities for genes located within the same TADs. This could be due to a lack of temporal coordination between multiple EPIs within the same TADs or between the EPI formation and the transcription activity of the corresponding gene [50, 57, 58]. Interestingly, we observed a TAD-dependent shift of the SCC distribution toward positive values for gene pairs located across the same TAD boundaries (Fig. 4f, g), indicating some mechanism temporally coordinate the transcription of genes across the same TAD boundaries (Fig. 4h).

Finally, we propose a model that TADs may have a role in modulating transcriptional burst frequency, likely via TAD-enhanced EPIs, leading to a smaller burst frequency variation for genes located within the same TADs (Fig. 4h).

## Discussion

Here, we developed LAST-seq as a new scRNA-seq method with its unique chemistry bypassing the inefficient RT/SSS prior to RNA amplification. Thanks to the direct linear amplification of the original ssRNA molecules in single cells, LAST-seq offers a high single-molecule RNA capture efficiency and a low level of technical noise for single-cell transcriptome analyses. LAST-seq’s superior capture efficiency benefits the study of gene expression noise and transcriptional bursting, enables the detection of minor RNA level differences between individual cells, and elevates the overall quality of single-cell transcriptome data. In addition, direct amplification of the original ssRNA molecules without prior RT/SSS provides an alternative for applications other than scRNA-seq, such as in situ RNA sequencing or low-quality RNA library preparation and sequencing, where the result may be improved by avoiding the inefficient RT/SSS on crosslinked or damaged RNA.

We evaluated the performance of LAST-seq against two representative scRNA-seq methods, SMART-seq as the most widely used method, and CEL-seq as another highly sensitive method based on linear amplification. For SMART-seq, we used its most advanced commercially available kit (SMART-seqV4) optimized from the chemistry of SMART-seq2 [5]. For CEL-seq, we used its most updated protocol of CEL-seq2 [7]. Recent development led to a new version of SMART-seq incorporating UMIs for the digital counting of RNA copies, SMART-seq3 [8], as well as its miniaturization to achieve a high cellular throughput, SMART-seq3xpress [20] and FLASH-seq [19], that are not widely available at the moment.

We estimate the reagent cost per cell for scRNA-seq to be similar between LAST-seq, SMART-seq, and CEL-seq, when performed in tubes or wells. In terms of the cellular throughput, the current version of LAST-seq is limited by the manual pipetting capability on 96-well or 384-well plates. We anticipate LAST-seq to be further optimized and miniaturized in microfluidic droplets, improving its performance while enhancing its cellular throughput, similar to the scaling-up efforts already made for RT/SSS-dependent scRNA-seq methods, such as the 10X Chromium instrument based on the chemistry of SMART-seq2 but with UMI attachment, the Drop-Seq technology [11] based on SMART-seq2, and the inDrop sequencing [10] based on CEL-seq.

In this study, LAST-seq was applied to profile the transcriptome of single human cells, followed by the estimation of transcriptional burst parameters of individual genes. Although a common practice, using the two-state model of transcriptional bursting to derive burst parameters from single-cell data is a simplification [59] and could lead to inaccuracies. Furthermore, when studying the role of TADs in modulating burst frequency for genes located with the same TADs, only those TADs containing enough number of genes with reliably estimated burst parameters were included in our analysis. As a result, the conclusion may not be generally applicable to all the TADs identified in the human genome, especially the ones containing few expressed genes were excluded. Future single-cell studies are needed to further elucidate the role of TADs in regulating transcriptional bursting and to investigate the observed temporal correlation of transcription activities for genes located across the same TAD boundaries. Single-cell RNA-seq with a reduced level of technical noise offered by LAST-seq, combined with a high cellular throughput, will be a useful approach to address these questions.

## Conclusions

LAST-seq is a new scRNA-seq method offering a high single-molecule RNA capture efficiency and detection sensitivity. Instead of performing RT/SSS before RNA amplification, LAST-seq directly and linearly amplifies the original ssRNA molecules in single cells without prior conversion to double-stranded DNA templates. Utilizing its reduced level of technical noise, we applied LAST-seq to study gene expression noise and transcriptional bursting in human cells, revealing a role of TADs in the regulation of transcriptional bursting and the temporal coordination between transcription activities.

## Methods

### Cell culture

HEK293T cells (ATCC) were cultured in complete DMEM medium (Gibco) containing 4.5 g/L glucose and 2 mM L-glutamine (Gibco), supplemented with 10% Fetal Bovine Serum (FBS) (Sigma-Aldrich) and 100 μg/ml penicillin–streptomycin (PS) (Gibco). The eHAP haploid human cells (Horizon discovery Ltd, Cat # C669) [37] were cultured in IMDM medium (Gibco) supplemented with 10% FBS and 1% PS. Upon subcloning, haploid human cells were enriched by flow-sorting based on cell size and cultured in the presence of 10 μM 10-Deacetylbaccatin-III (Selleckchem) to maintain the haploid status [60]. HEK293T was not authenticated. eHAP haploid human cell line was authenticated by DNA content staining.

### Gene editing

The crRNA targeting *SCC4* (Additional file 2: Table S2) was designed by the IDT Alt-R Custom Cas9 crRNA Design Tool and annealed to the tracrRNA to form the 100 μM crRNA::tracrRNA duplex, followed by a 20-min room-temperature incubation to assemble with the purified Cas9 nuclease (IDT) in a 5 μl reaction volume consisting of 2.1 μl PBS, 1.2 μl crRNA::tracrRNA duplex, and 1.7 μl Cas9 nuclease. The resulting Cas9/gRNA complex was then electroporated into 0.5 million eHAP cells by the SE Cell Line 4D-NucleofectorTM X Kit L (Lonza) using the built-in EN-138 program. After a 48-h incubation, successful *SCC4* knockout (KO) was detected by the genome-editing detection kit (IDT). Single haploid human cells were then flow-sorted based on cell size into 96-well plates and cultured for 10 days for single-cell clonal expansion. PCR genotyping was then performed using half of the cells from each clonal population, with verified *SCC4* KO clones further cultured in 6-well plates, before the final validation of the successful KO by Western blot.

### PCR genotyping

Cells were washed in PBS and resuspended in 10 μl QuickExtract DNA Extraction Solution (Lucigen). The cell lysate was then incubated at 65 °C for 10 min, 98 °C for 5 min, followed by PCR using Q5 High-Fidelity Master Mix and 0.5 μM corresponding primers (Additional file 2: Table S2). The PCR products were analyzed by 10% DNA polyacrylamide gel electrophoresis.

### Western blot

Cells were washed in PBS and lysed in 30 μl RIPA buffer (Thermo Fisher Scientific) containing protease inhibitor cocktail (Sigma-Aldrich). The cell lysate was then mixed with 10 μl Laemmli Sample Buffer (Bio-Rad) for a 5-min 95°C incubation, followed by 10% SDS-PAGE and transfer to nitrocellulose membrane for immunoblotting analysis. Anti-Scc4 antibody (Abcam, ab183033) and anti-beta actin antibody (Abcam, ab8227) were used as primary antibodies, and Goat Anti-Rabbit IgG H&L (Abcam, ab6721) was used as the secondary antibody.

### Preparing single-stranded RNA templates for T7 IVT

To make short ssRNA templates for T7 IVT, 50 μM each of sense strands and anti-sense strands (Additional file 2: Table S2) were mixed in equal molar number in the annealing buffer (20 mM Tris-Ac pH 8.3, 50 mM NaCl, 2 mM EDTA pH 8.0), incubated at 95°C for 3 min, and slow cooled down to 25°C. To make long ssRNA templates for IVT, dsDNA templates containing a T7 and T3 promoter with a 30-nt poly-T element were cloned from the PUC18 (template for T1.2K) and LD220 (template for T2.2K), respectively (Addgene). RNAs were then generated by IVT of the dsDNA templates, followed by DNase I treatment and RNA column purification (NEB), and finally annealed to the linear primer (Additional file 2: Table S2) by a 5-min 70°C denaturation step and immediate cool-down on ice. After the RNA column purification, the annealed products were converted to the final long ssRNA templates for T7 IVT, by a 1-h 37°C incubation with RNase H and Klenow Fragment exo^−^ in the presence of dNTP and RNase Inhibitor, followed by column purification. To make transcriptomic ssRNA templates for IVT, total RNA extracted from the HEK293T cells was annealed to the linear primer and converted to the final ssRNA templates, following the same procedure used for the preparation of long ssRNA templates for T7 IVT.

### IVT of single-stranded RNA templates

To perform IVT on ssRNA templates, 10 ng ssRNA templates were mixed with 20 μl IVT solution (1X RNA Pol Reaction Buffer, 2.5 mM NTPs, 5 U/μl T7 RNA polymerase, 1 U/μl RNase Inhibitor, 0.01% Triton X-100) for incubation at 37°C for up to 16 h. IVT products were RNA column purified, DNase I treated, and resolved by 10% urea PAGE or 1.5% agarose denaturing gel electrophoresis, or quantified by RT-qPCR using corresponding primers (Additional file 2: Table S2) and the Verso 1-step RT-qPCR Kit (Thermo Fisher Scientific) on the QuantStudio 5 Real-Time PCR System (Applied Biosystems) with the Delta-Delta Ct method. The assembled IVT reaction without any incubation was used as the negative control.

### Total RNA and genomic DNA extraction

Total RNA was extracted from HEK293T cells using the Total RNA Miniprep Kit (NEB) with an on-column DNase digestion step. Genomic DNA was extracted from HEK293T cells using the genomic DNA purification Kit (NEB). The total RNA and genomic DNA were Nanodrop quantified. RNA integrity was determined using the Agilent 2100 Bioanalyzer.

### Synthesis and digital quantification of the A60 RNA spike-in species

Specific regions of 24 ERCC spike-ins with a T7 promoter attachment were synthesized (IDT) and amplified using a forward primer annealing to the T7 promoter and a reverse primer containing 60 Ts (Additional file 2: Table S4). The resulting dsDNA templates were subject to T7 IVT, and the transcribed RNAs were quantified using the Qubit RNA HS Assay Kit (Invitrogen) and the Bioanalyzer RNA 6000 Pico Kit (Agilent). The 24 RNA spike-in species were then diluted and mixed into the A60 RNA spike-in mix (Additional file 2: Table S5). For digital quantification, the A60 RNA spike-in mix was converted to cDNA by oligo-dT_60_ primed reverse transcription and quantified by the QX200 Droplet Digital (ddPCR) system using the QX200 EvaGreen Supermix (Bio-Rad), Droplet Generation Oil (Bio-Rad), and the corresponding primers (Additional file 2: Table S4). The copy number of each A60 RNA spike-in species is reported in Additional file 2: Table S5.

### Single-cell isolation

Single-cell suspensions of HEK293T cells and eHAP human haploid cells were made by trypsin dissociation. HEK293T cells were stained by the Zombie Green dye (BioLegend) to exclude dead cells. The eHAP cells were stained by both 10 μg/ml Hoechst33342 (Invitrogen) and the Zombie Green dye at 37 °C for 15 min to label live haploid and G1-phase cells. Single cells were then flow-sorted into 96-well plates with 2 μl lysis buffer in each well, using the BD FACSAria Fusion.

### LAST-seq assay

#### Cellular barcode design

The 6-nt cellular barcodes were designed using the DNABarcodes R package [61] allowing a minimal hamming distance of 3. Barcodes that anneal to the T7 promoter sequence were also eliminated. 80 cellular barcodes were selected (Additional file 2: Table S3).

#### Preparing the LAST-seq primer

The LAST-seq primer was made by the ligation between a hairpin module and an rU-dT module. The hairpin module consists of a looping region, a T7 promoter, a PCR handle, and the UMI and cellular barcode (CB) region. The rU-dT module consists of a complementary linker and an rU-dT tail with a 3′-end dideoxycytidine (ddC) blocker (Additional file 1: Fig. S3a). Following a heating and cooling program for the hairpin module to form a loop itself, the hairpin module was annealed and ligated with the rU-dT module at 16°C for 30 min in a 20 μl E. coli DNA ligase reaction (Additional file 1: Fig. S3b). The ligation product was then purified by 10% urea polyacrylamide gel electrophoresis (Additional file 1: Fig. S3c). Specifically, a small piece of polyacrylamide gel containing the ligation product was soaked in 400 μl 300 μM NaOAc, incubated on dry ice for 15 min and at room temperature for 4 h with a rotator, and ethanol precipitated. Finally, the purified LAST-seq primer was quantified by Nanodrop.

#### Single-cell lysis

Single cells were flow-sorted into 96-well plates with 2 μl lysis buffer in each well, containing 0.1% Triton X-100, 2 nM LAST-seq primer, 0.1 U/μl RNase Inhibitor, 0.5 mM dNTP, 60 pg RNA carriers, and the RNA spike-in (1 μl 1:1000,000 diluted ERCC spike-in or 1:20,000 diluted A60 spike-in). The plate was then sealed with aluminum film (MilliporeSigma), briefly spun down, and lysed at 65°C for 5 min before placing on ice.

#### Linear amplification of the single-stranded RNA molecules by T7 IVT

The single-cell lysate was evaporated at 30°C for 25 min by Eppendorf Vacufuge, resuspended in 10 μl (for 10 cells pooling) or 2.5 μl (for 80 cells pooling) buffer containing 1X NEBuffer2, 0.3 mM dNTP, 1 U/μl RNase Inhibitor, 0.02 U/μl RNase H, and 0.3 U/μl Klenow Fragment exo^−^, incubated at 37°C for 30 min and then kept on ice. Single-cell lysates from 10 or 80 cells were then pooled into one DNA LoBind tube and purified by 0.5X beads (Aline Bioscience, C-1005–5/50) and 1.3X buffer. Next, the beads were rehydrated in 25 μl T7 IVT buffer containing T7 RNAPol Reaction Buffer, 1.25 mM NTP, 1 U/μl RNase Inhibitor, 5 mM DTT, 5 mM MgCl_2_ and 2.5 U/μl T7 RNA Polymerase. After incubation overnight at 37°C for up to 16 h, the aRNA products were purified using 0.8X beads and 1X buffer, with final elution using 7 μl of nuclease-free water.

#### Library preparation of the amplified products

Six microliters of aRNA products were mixed with 0.5 μl 10 mM dNTP and 0.5 μl 25 mM random primers, heated at 70°C for 2 min and cooled on ice, assembled into a reverse transcription reaction containing 2 μl 5X first-strand synthesis buffer, 0.25 μl 20 U/μl RNase Inhibitor, 0.25 μl 0.4 M DTT and 0.5 μl 200 U/μl SuperScript IV (Invitrogen), and incubated at 23°C for 10 min, 50°C for 15 min, 80°C for 10 min. The RT products were PCR amplified for 8 cycles by the Q5 High-Fidelity Master Mix and P5/P7 primers, followed by 0.9X beads purification (Aline Bioscience). The purification products were further PCR amplified, beads purified, and eluted in 11 μl 0.1 × TE to get the final library for Illumina sequencing (Additional file 1: Fig. S8).

## SMART-seq

Single-cell mRNAs were amplified by the SMART-seqV4 ultra-low input RNA kit (Takara) according to the user manual. Briefly, the single-cell or single-cell lysate was distributed into individual PCR tubes containing 5 μl PBS. The lysis buffer containing spike-ins was added to the tube, followed by heating and cooling for single-cell lysis. mRNAs were converted into cDNA by the SMARTScribe reverse transcriptase and were amplified by 17 cycles of PCR. Libraries were generated using the NEBNext Ultra II DNA library prep kit.

## Calculation of the detection rate

LAST-seq and SMART-seq were applied to single-cell lysate containing 1 μl ERCC (1:1,000,000) spike-in. For each spike-in species, we determined whether it was detected or not for each well. The ratio of “detected wells” to “total wells” was calculated as the detection rate. The sliding window strategy (zoo v.1.8.9 [62]; window size = 9; step = 1) was used to smoothen the data by taking a sliding mean of the detection rates.

## CEL-seq

CEL-seq libraries were generated in accordance with the CEL-seq2 protocol [7]. Briefly, 10 single cells or cell lysates were distributed into 10 PCR tubes containing 1.2 μl lysis buffer with 0.5 μl 1:500,000 diluted ERCC spike-in or 1:10,000 diluted A60 spike-in. PCR tubes were heated at 65 °C for 5 min and quickly cooled on ice to lyse cells and anneal to the poly-A tail. After reverse transcription and second-strand synthesis, the cDNA from the 10 cells was pooled, bead purified, and in vitro transcribed for 13 h. The IVT products were then fragmented by heating and converted to cDNA by reverse transcription, followed by 15 cycles of PCR amplification and 2 rounds of bead purification.

## Genomic DNA contamination

Total RNA and genomic DNA were extracted from HEK293T cells. LAST-seq and CEL-seq were performed on 100 pg total RNA and 60 pg genomic DNA, following the protocols above. For LAST-seq, 13 cycles of PCR were used to amplify the final libraries from total RNA and genomic DNA. All libraries were quantified by Qubit dsDNA HS Assay Kit (Invitrogen). The library yield was presented in Additional file 2: Table S1.

## Calculation of minimum free energy

The cDNA sequences of selected genes were downloaded from the Ensembl database by BioMart. ViennaRNA [63] and customized scripts were used to calculate and extract the minimum free energy, respectively.

## Sequencing

Libraries were sequenced on an Illumina NextSeq 550 instrument with a High Output v2.5 reagent kit (Illumina). For LAST-seq and SMART-seqV4 libraries, read1 was sequenced for 50 bases, and read2 was sequenced for 25 bases. For CEL-seq libraries, read1 was sequenced for 25 bases, and read2 was sequenced for 50 bases.

## Sequencing data processing

The sequencing data was converted to fastq format by bcl2fastq v2.20, followed by reads trimming using cutadapt v 1.15 [64] with parameters ‘–nextseq-trim = 20 -m 22:14’. The zUMIs v.2.9.7 [65] pipeline was used for alignment (GRCh38.p13/annotationv33 and ERCC spike-in reference), subsampling, and reads/UMI counting. Reads with more than one base below Phred 20 base call scores in the UMI sequence were discarded. UMIs were collapsed by sequence identity. Reads were subsampled to an equal depth for data of LAST-seq, SMART-seq, and CEL-seq. Reads mapped to exons were used for downstream analysis.

## Modeling the technical noise of LAST-seq

Given the single-molecule capture efficiency of LAST-seq, the quantification of mRNA level (X) observes the binomial distribution (B) as follows:

$$X\sim B\left(n,p\right)$$

where *n* denotes the original mRNA level, and *p* denotes capture efficiency of a single mRNA molecule.

The coefficient of variation (CV) represents the technical noise which was computed as follows:

$$CV=\sqrt{(1-p)/np}$$

where *np* equals the observed expression level on average. We assumed *p* = 0.35 as the capture efficiency to generate the plot.

## Transcriptional bursting kinetics

Single haploid human cells (eHAP) were sequenced to an average depth of ~ 4.6 M reads per cell. Empty wells (exon reads < 50,000) and wells with potential contamination (exon mapping rate < 50%) were eliminated. One hundred forty-eight WT and 153 *SCC4* KO eHAP cells were used for downstream analysis. After the batch effect removal by ComBat-seq [66], an established script [33] (https://github.com/sandberg-lab/txburst) was used to infer the parameters of transcriptional bursting kinetics from the single-cell RNA-seq data. Specifically, the two-state model of transcriptional bursting was used to simulate the theoretical distribution of the mRNA copy number in individual cells for each gene. After reaching the steady state between transcriptional bursting and mRNA degradation, the theoretical distribution of the mRNA copy number for each gene in single cells, captured at a time snapshot by the scRNA-seq assay, will observe a Poisson-Beta distribution. The parameters of the Poisson-Beta distribution will reflect transcriptional bursting parameters including *K*_on_, *K*_off_, and *K*_syn_. As a result, bursting parameters could be derived by fitting the mRNA copy number distribution of each gene observed by single-cell RNA-seq into the theoretical Poisson-Beta distribution, using the maximum likelihood inference to generate the best estimates of transcriptional bursting parameters *K*_on_, *K*_off_, and *K*_syn_ for each gene. Since all the parameters were estimated in the unit of a constant mRNA degradation rate, the final burst frequency (*K*_on_) is still in the unit of the mRNA degradation rate while the final burst size (*K*_syn_/*K*_on_) is not. Since the variability in the mRNA degradation rates of individual genes does not contribute much to the estimated bursting parameters [33], a universal mRNA degradation rate constant was used in our estimation. The estimated bursting parameters are already relatively stable when use the single-cell transcriptome data from a random half of the sequenced cells, suggesting enough statistical power to estimate bursting parameters given the cell numbers used in our study. Finally, transcriptional bursting parameters were estimated for 5156 genes in the WT haploid human cells and 4435 genes in the *SCC4* KO haploid human cells (Additional file 1: Fig. S6g).

## Hi-C data analysis

The processed haploid human cell Hi-C contact matrix and A/B compartment data were downloaded from the 4D Nucleome Data Portal, contributed by Erez Lieberman Aiden Lab [67]. TADs were called by HiCExplorer3.7.2 [68] at 100 K bin size resolution. The TAD boundary was defined as the 5% region of the two neighboring TADs spanning across the boundary. The A/B compartment data was converted to the bedgraph format by bigWigToBedGraph from UCSC. Gene coordinates were compared to the location of TADs, TAD boundaries, and A/B compartments by BEDOPS v2.4.40 [69]. Genes completely covered by those 3D genomic features were used for downstream analysis. To compare the Hi-C matrix between the WT cells and *SCC4* KO cells, data from Benjamin D. Rowland lab [55] was visualized by JuiceBox [70].

## Comparison of expression levels

To evaluate the transcriptomic changes after *SCC4* knockout, the R package BASiCS [71] was used to identify genes with differential expression level. Genes with < 0.1 mean expression level were excluded. A 5% expected FDR was used for the statistical test.

## Coefficient of variation (CV) of burst frequency

To compare the burst frequency variation of genes in the context of TADs, we calculated the CV of burst frequency for groups of genes located within the same TAD or across the same TAD boundary. The CV was calculated by:

$$CV=\frac{\upsigma }{\upmu }$$

where $\sigma$ denotes the standard deviation, and $\mu$ denotes the mean.

In WT eHAP1 cells, CVs were computed in 59 TADs, each containing at least 8 genes, and 37 TAD boundaries, each containing at least 4 genes. In *SCC4* KO cells, CVs were computed in 37 TADs and 32 TAD boundaries, under the same criterion of gene number. For the control of randomly picked genes across the transcriptome, we computed the CV for a random group of genes, with the group size equal to the median gene number in the selected TADs (10 genes) or TAD boundaries (5 genes). Finally, to compare the CVs of burst frequency for genes located within TADs, across TAD boundaries, and randomly picked control, in both WT and *SCC4* KO cells, we performed 10,000 bootstraps and plotted the distribution of the median value of the CVs for comparison.

## Calculation of the Spearman’s rank correlation coefficient between gene pairs

Genes with extremely low expression levels (< 2 transcripts per cell on average) were excluded. The transcript level of each gene was normalized by the total number of UMIs for each individual cell, in order to normalize the global cell-to-cell variation due to the residue extrinsic noise. Correlation coefficients between the mRNA transcript levels of gene pairs were calculated, for all gene pairs located in TADs and TAD boundaries containing at least a pair of genes. For the control, correlation coefficients were calculated between the mRNA levels of gene pairs randomly selected from the transcriptome. In WT eHAP1 cells, correlation coefficients were computed between 16712871, 6711, and 987 gene pairs, for the control, for genes in 551 TADs, and for genes across 249 TAD boundaries, respectively. In *SCC4KO* cells, correlation coefficients were computed between 17508403, 7297, and 1064 gene pairs, for the control, for genes in 560 TADs, and for genes across 250 TAD boundaries, respectively.

### Supplementary Information

**Additional file 1:** **Fig. S1.** Simulated performance of scRNA-seq with varying single-molecule capture efficiency. **Fig. S2.** T7 In vitro transcription (IVT) of ssRNA templates. **Fig. S3.** LAST-seq primer. **Fig. S4.** Performance of LAST-seq and comparison to SMART-seq. **Fig. S5.** Comparison between LAST-seq and CEL-seq. **Fig. S6.** Transcriptional bursting kinetics in human cells. **Fig. S7.** Chromatin structure and gene expression level in wildtype and SCC4 knockout haploid human cells. **Fig. S8.** LAST-seq library.

**Additional file 2:** **Table S1.** gDNA contamination test. **Table S2.** Oligos of short IVT templates; primers for long template construction and IVT quantification; primer for SCC4 knockout. **Table S3.** LAST-seq barcoding primers and library indexing primers. **Table S4.** Primers for A60 spike-ins. **Table S5.** Quantification of A60 spike-ins.

**Additional file 3.** Review history.

## Data Availability

The sequencing data were deposited at the National Center for Biotechnology Information Gene Expression Omnibus (GEO), with the accession number GSE211836 [72]. The code required to produce the main figures and findings is available at https://github.com/lyuj2022/LAST-seq [73] (published under MIT License). A stable version of the github repository is available through zenodo (10.5281/zenodo.8169902) [74].

## References

[1] Tang F, Barbacioru C, Wang Y, Nordman E, Lee C, Xu N, Wang X, Bodeau J, Tuch BB, Siddiqui A (2009). mRNA-Seq whole-transcriptome analysis of a single cell. Nat Methods.

[2] Islam S, Kjallquist U, Moliner A, Zajac P, Fan JB, Lonnerberg P, Linnarsson S (2011). Characterization of the single-cell transcriptional landscape by highly multiplex RNA-seq. Genome Res.

[3] Ramskold D, Luo S, Wang YC, Li R, Deng Q, Faridani OR, Daniels GA, Khrebtukova I, Loring JF, Laurent LC (2012). Full-length mRNA-Seq from single-cell levels of RNA and individual circulating tumor cells. Nat Biotechnol.

[4] Hashimshony T, Wagner F, Sher N, Yanai I (2012). CEL-Seq: single-cell RNA-Seq by multiplexed linear amplification. Cell Rep.

[5] Picelli S, Bjorklund AK, Faridani OR, Sagasser S, Winberg G, Sandberg R (2013). Smart-seq2 for sensitive full-length transcriptome profiling in single cells. Nat Methods.

[6] Islam S, Zeisel A, Joost S, La Manno G, Zajac P, Kasper M, Lonnerberg P, Linnarsson S (2014). Quantitative single-cell RNA-seq with unique molecular identifiers. Nat Methods.

[7] Hashimshony T, Senderovich N, Avital G, Klochendler A, de Leeuw Y, Anavy L, Gennert D, Li S, Livak KJ, Rozenblatt-Rosen O (2016). CEL-Seq2: sensitive highly-multiplexed single-cell RNA-Seq. Genome Biol.

[8] Hagemann-Jensen M, Ziegenhain C, Chen P, Ramskold D, Hendriks GJ, Larsson AJM, Faridani OR, Sandberg R (2020). Single-cell RNA counting at allele and isoform resolution using Smart-seq3. Nat Biotechnol.

[9] Jaitin DA, Kenigsberg E, Keren-Shaul H, Elefant N, Paul F, Zaretsky I, Mildner A, Cohen N, Jung S, Tanay A, Amit I (2014). Massively parallel single-cell RNA-Seq for marker-free decomposition of tissues into cell types. Science.

[10] Klein AM, Mazutis L, Akartuna I, Tallapragada N, Veres A, Li V, Peshkin L, Weitz DA, Kirschner MW (2015). Droplet barcoding for single-cell transcriptomics applied to embryonic stem cells. Cell.

[11] Macosko EZ, Basu A, Satija R, Nemesh J, Shekhar K, Goldman M, Tirosh I, Bialas AR, Kamitaki N, Martersteck EM (2015). Highly parallel genome-wide expression profiling of individual cells using nanoliter droplets. Cell.

[12] Yuan J, Sims PA (2016). An automated microwell platform for large-scale single cell RNA-Seq. Sci Rep.

[13] Zheng GX, Terry JM, Belgrader P, Ryvkin P, Bent ZW, Wilson R, Ziraldo SB, Wheeler TD, McDermott GP, Zhu J (2017). Massively parallel digital transcriptional profiling of single cells. Nat Commun.

[14] Gierahn TM, Wadsworth MH, Hughes TK, Bryson BD, Butler A, Satija R, Fortune S, Love JC, Shalek AK (2017). Seq-Well: portable, low-cost RNA sequencing of single cells at high throughput. Nat Methods.

[15] Cao JY, Packer JS, Ramani V, Cusanovich DA, Huynh C, Daza R, Qiu X, Lee C, Furlan SN, Steemers FJ (2017). Comprehensive single-cell transcriptional profiling of a multicellular organism. Science.

[16] Rosenberg AB, Roco CM, Muscat RA, Kuchina A, Sample P, Yao ZZ, Graybuck LT, Peeler DJ, Mukherjee S, Chen W (2018). Single-cell profiling of the developing mouse brain and spinal cord with split-pool barcoding. Science.

[17] Han X, Wang R, Zhou Y, Fei L, Sun H, Lai S, Saadatpour A, Zhou Z, Chen H, Ye F (2018). Mapping the mouse cell atlas by Microwell-seq. Cell.

[18] Sheng K, Cao W, Niu Y, Deng Q, Zong C (2017). Effective detection of variation in single-cell transcriptomes using MATQ-seq. Nat Methods.

[19] Hahaut V, Pavlinic D, Carbone W, Schuierer S, Balmer P, Quinodoz M, Renner M, Roma G, Cowan CS, Picelli S (2022). Fast and highly sensitive full-length single-cell RNA sequencing using FLASH-seq. Nat Biotechnol.

[20] Hagemann-Jensen M, Ziegenhain C, Sandberg R (2022). Scalable single-cell RNA sequencing from full transcripts with Smart-seq3xpress. Nat Biotechnol.

[21] Salmen F, De Jonghe J, Kaminski TS, Alemany A, Parada GE, Verity-Legg J, Yanagida A, Kohler TN, Battich N, van den Brekel F (2022). High-throughput total RNA sequencing in single cells using VASA-seq. Nat Biotechnol.

[22] Chapman AR, He Z, Lu S, Yong J, Tan L, Tang F, Xie XS (2015). Single cell transcriptome amplification with MALBAC. PLoS ONE.

[23] Di L, Liu B, Lyu Y, Zhao S, Pang Y, Zhang C, Wang J, Qi H, Shen J, Huang Y (2022). Rapid and sensitive single-cell RNA sequencing with SHERRY2. BMC Biol.

[24] Van Gelder RN, von Zastrow ME, Yool A, Dement WC, Barchas JD, Eberwine JH (1990). Amplified RNA synthesized from limited quantities of heterogeneous cDNA. Proc Natl Acad Sci U S A.

[25] Chen C, Xing D, Tan L, Li H, Zhou G, Huang L, Xie XS (2017). Single-cell whole-genome analyses by Linear Amplification via Transposon Insertion (LIANTI). Science.

[26] Arnaud-Barbe N, Cheynet-Sauvion V, Oriol G, Mandrand B, Mallet F (1998). Transcription of RNA templates by T7 RNA polymerase. Nucleic Acids Res.

[27] Duftner N, Larkins-Ford J, Legendre M, Hofmann HA (2008). Efficacy of RNA amplification is dependent on sequence characteristics: implications for gene expression profiling using a cDNA microarray. Genomics.

[28] Mereu E, Lafzi A, Moutinho C, Ziegenhain C, McCarthy DJ, Alvarez-Varela A, Batlle E, Sagar, Grun D, Lau JK, et al: Benchmarking single-cell RNA-sequencing protocols for cell atlas projects. Nat Biotechnol 2020, 38:747–755. 10.1038/s41587-020-0469-4 32518403

[29] Baker SC, Bauer SR, Beyer RP, Brenton JD, Bromley B, Burrill J, Causton H, Conley MP, Elespuru R, Fero M (2005). The external RNA controls consortium: a progress report. Nat Methods.

[30] Legnini I, Alles J, Karaiskos N, Ayoub S, Rajewsky N (2019). FLAM-seq: full-length mRNA sequencing reveals principles of poly(A) tail length control. Nat Methods.

[31] Svensson V, Natarajan KN, Ly LH, Miragaia RJ, Labalette C, Macaulay IC, Cvejic A, Teichmann SA (2017). Power analysis of single-cell RNA-sequencing experiments. Nat Methods.

[32] La Manno G, Soldatov R, Zeisel A, Braun E, Hochgerner H, Petukhov V, Lidschreiber K, Kastriti ME, Lonnerberg P, Furlan A (2018). RNA velocity of single cells. Nature.

[33] Larsson AJM, Johnsson P, Hagemann-Jensen M, Hartmanis L, Faridani OR, Reinius B, Segerstolpe A, Rivera CM, Ren B, Sandberg R (2019). Genomic encoding of transcriptional burst kinetics. Nature.

[34] Li C, Cesbron F, Oehler M, Brunner M, Hofer T (2018). Frequency modulation of transcriptional bursting enables sensitive and rapid gene regulation. Cell Syst.

[35] Lenstra TL, Rodriguez J, Chen H, Larson DR (2016). Transcription dynamics in living cells. Annu Rev Biophys.

[36] Reinius B, Mold JE, Ramskold D, Deng Q, Johnsson P, Michaelsson J, Frisen J, Sandberg R (2016). Analysis of allelic expression patterns in clonal somatic cells by single-cell RNA-seq. Nat Genet.

[37] Essletzbichler P, Konopka T, Santoro F, Chen D, Gapp BV, Kralovics R, Brummelkamp TR, Nijman SM, Burckstummer T (2014). Megabase-scale deletion using CRISPR/Cas9 to generate a fully haploid human cell line. Genome Res.

[38] Rao SS, Huntley MH, Durand NC, Stamenova EK, Bochkov ID, Robinson JT, Sanborn AL, Machol I, Omer AD, Lander ES, Aiden EL (2014). A 3D map of the human genome at kilobase resolution reveals principles of chromatin looping. Cell.

[39] Dey SS, Foley JE, Limsirichai P, Schaffer DV, Arkin AP (2015). Orthogonal control of expression mean and variance by epigenetic features at different genomic loci. Mol Syst Biol.

[40] Spielmann M, Lupianez DG, Mundlos S (2018). Structural variation in the 3D genome. Nat Rev Genet.

[41] Krefting J, Andrade-Navarro MA, Ibn-Salem J (2018). Evolutionary stability of topologically associating domains is associated with conserved gene regulation. BMC Biol.

[42] Finn EH, Misteli T (2019). A genome disconnect. Nat Genet.

[43] Ghavi-Helm Y, Jankowski A, Meiers S, Viales RR, Korbel JO, Furlong EEM (2019). Highly rearranged chromosomes reveal uncoupling between genome topology and gene expression. Nat Genet.

[44] Rao SSP, Huang SC, Glenn St Hilaire B, Engreitz JM, Perez EM, Kieffer-Kwon KR, Sanborn AL, Johnstone SE, Bascom GD, Bochkov ID (2017). Cohesin loss eliminates all loop domains. Cell.

[45] Cuartero S, Weiss FD, Dharmalingam G, Guo Y, Ing-Simmons E, Masella S, Robles-Rebollo I, Xiao X, Wang YF, Barozzi I (2018). Control of inducible gene expression links cohesin to hematopoietic progenitor self-renewal and differentiation. Nat Immunol.

[46] Stik G, Vidal E, Barrero M, Cuartero S, Vila-Casadesus M, Mendieta-Esteban J, Tian TV, Choi J, Berenguer C, Abad A (2020). CTCF is dispensable for immune cell transdifferentiation but facilitates an acute inflammatory response. Nat Genet.

[47] Long HK, Prescott SL, Wysocka J (2016). Ever-changing landscapes: transcriptional enhancers in development and evolution. Cell.

[48] McCord RP, Kaplan N, Giorgetti L (2020). Chromosome conformation capture and beyond: toward an integrative view of chromosome structure and function. Mol Cell.

[49] Eling N, Morgan MD, Marioni JC (2019). Challenges in measuring and understanding biological noise. Nat Rev Genet.

[50] Xiao JY, Hafner A, Boettiger AN (2021). How subtle changes in 3D structure can create large changes in transcription. Elife.

[51] Bartman CR, Hsu SC, Hsiung CC, Raj A, Blobel GA (2016). Enhancer regulation of transcriptional bursting parameters revealed by forced chromatin looping. Mol Cell.

[52] Fukaya T, Lim B, Levine M (2016). Enhancer control of transcriptional bursting. Cell.

[53] Ringel AR, Szabo Q, Chiariello AM, Chudzik K, Schopflin R, Rothe P, Mattei AL, Zehnder T, Harnett D, Laupert V (2022). Repression and 3D-restructuring resolves regulatory conflicts in evolutionarily rearranged genomes. Cell.

[54] Nicolas D, Phillips NE, Naef F (2017). What shapes eukaryotic transcriptional bursting?. Mol Biosyst.

[55] Haarhuis JHI, van der Weide RH, Blomen VA, Yanez-Cuna JO, Amendola M, van Ruiten MS, Krijger PHL, Teunissen H, Medema RH, van Steensel B (2017). The cohesin release factor WAPL restricts chromatin loop extension. Cell.

[56] Hsieh TS, Cattoglio C, Slobodyanyuk E, Hansen AS, Darzacq X, Tjian R (2022). Enhancer-promoter interactions and transcription are largely maintained upon acute loss of CTCF, cohesin, WAPL or YY1. Nat Genet.

[57] Alexander JM, Guan J, Li B, Maliskova L, Song M, Shen Y, Huang B, Lomvardas S, Weiner OD (2019). Live-cell imaging reveals enhancer-dependent Sox2 transcription in the absence of enhancer proximity. Elife.

[58] Zuin J, Roth G, Zhan Y, Cramard J, Redolfi J, Piskadlo E, Mach P, Kryzhanovska M, Tihanyi G, Kohler H (2022). Nonlinear control of transcription through enhancer-promoter interactions. Nature.

[59] Zoller B, Nicolas D, Molina N, Naef F (2015). Structure of silent transcription intervals and noise characteristics of mammalian genes. Mol Syst Biol.

[60] Olbrich T, Vega-Sendino M, Murga M, de Carcer G, Malumbres M, Ortega S, Ruiz S, Fernandez-Capetillo O (2019). A chemical screen identifies compounds capable of selecting for haploidy in mammalian cells. Cell Rep.

[61] Buschmann T (2017). DNABarcodes: an R package for the systematic construction of DNA sample tags. Bioinformatics.

[62] Zeileis A, Grothendieck G: zoo: S3 infrastructure for regular and irregular time series. Journal of Statistical Software 2005, 14.

[63] Lorenz R, Bernhart SH (2011). Höner zu Siederdissen C, Tafer H, Flamm C, Stadler PF, Hofacker IL: ViennaRNA package 2.0. Algorithms Mol Biol.

[64] Martin M (2011). Cutadapt removes adapter sequences from high-throughput sequencing reads. EMBnetjournal.

[65] Parekh S, Ziegenhain C, Vieth B, Enard W, Hellmann I (2018). zUMIs - a fast and flexible pipeline to process RNA sequencing data with UMIs. Gigascience.

[66] Zhang Y, Parmigiani G, Johnson WE (2020). ComBat-seq: batch effect adjustment for RNA-seq count data. NAR Genom Bioinform.

[67] Sanborn AL, Rao SS, Huang SC, Durand NC, Huntley MH, Jewett AI, Bochkov ID, Chinnappan D, Cutkosky A, Li J (2015). Chromatin extrusion explains key features of loop and domain formation in wild-type and engineered genomes. Proc Natl Acad Sci U S A.

[68] Ramirez F, Bhardwaj V, Arrigoni L, Lam KC, Gruning BA, Villaveces J, Habermann B, Akhtar A, Manke T (2018). High-resolution TADs reveal DNA sequences underlying genome organization in flies. Nat Commun.

[69] Neph S, Kuehn MS, Reynolds AP, Haugen E, Thurman RE, Johnson AK, Rynes E, Maurano MT, Vierstra J, Thomas S (2012). BEDOPS: high-performance genomic feature operations. Bioinformatics.

[70] Robinson JT, Turner D, Durand NC, Thorvaldsdottir H, Mesirov JP, Aiden EL (2018). Juicebox.js provides a cloud-based visualization system for Hi-C data. Cell Systems.

[71] Vallejos CA, Marioni JC, Richardson S (2015). BASiCS: Bayesian analysis of single-cell sequencing data. Plos Computatl Biol.

[72] Lyu, J. Chen, C.: LAST-seq: single-cell RNA sequencing by direct amplification of single-stranded RNA without prior reverse transcription and second-strand synthesis. Datasets Gene Expression Omnibus. https://www.ncbi.nlm.nih.gov/geo/query/acc.cgi?acc=GSE211836. 2023. 10.1186/s13059-023-03025-5 PMC10413806 37559123

[73] Lyu, J. Chen, C.: LAST-seq: single-cell RNA sequencing by direct amplification of single-stranded RNA without prior reverse transcription and second-strand synthesis. Github https://github.com/lyuj2022/LAST-seq. 2023. 10.1186/s13059-023-03025-5 PMC10413806 37559123

[74] Lyu J, Chen C. LAST-seq: single-cell RNA sequencing by direct amplification of single-stranded RNA without prior reverse transcription and second-strand synthesis. Zenodo 10.5281/zenodo.8169902. 2023. 10.1186/s13059-023-03025-5 PMC10413806 37559123

