# Supplementary material for: LAST-seq: single-cell RNA sequencing by direct amplification of single-stranded RNA without prior reverse transcription and second-strand synthesis
Source: Genome Biol. 2023 Aug 9;24:184. doi: 10.1186/s13059-023-03025-5 (PMC10413806; doi:10.1186/s13059-023-03025-5)
Supplement: Supplementary file 1 — Additional file 1: Fig. S1. Simulated performance of scRNA-seq with varying single-molecule capture efficiency. Fig. S2. T7 In vitro transcription (IVT) of ssRNA templates. Fig. S3. LAST-seq primer. Fig. S4. Performance of LAST-seq and comparison to SMART-seq. Fig. S5. Comparison between LAST-seq and CEL-seq. Fig. S6. Transcriptional bursting kinetics in human cells. Fig. S7. Chromatin structure and gene expression level in wildtype and SCC4 knockout haploid human cells. Fig. S8. LAST-seq library. [file 13059_2023_3025_MOESM1_ESM.docx]

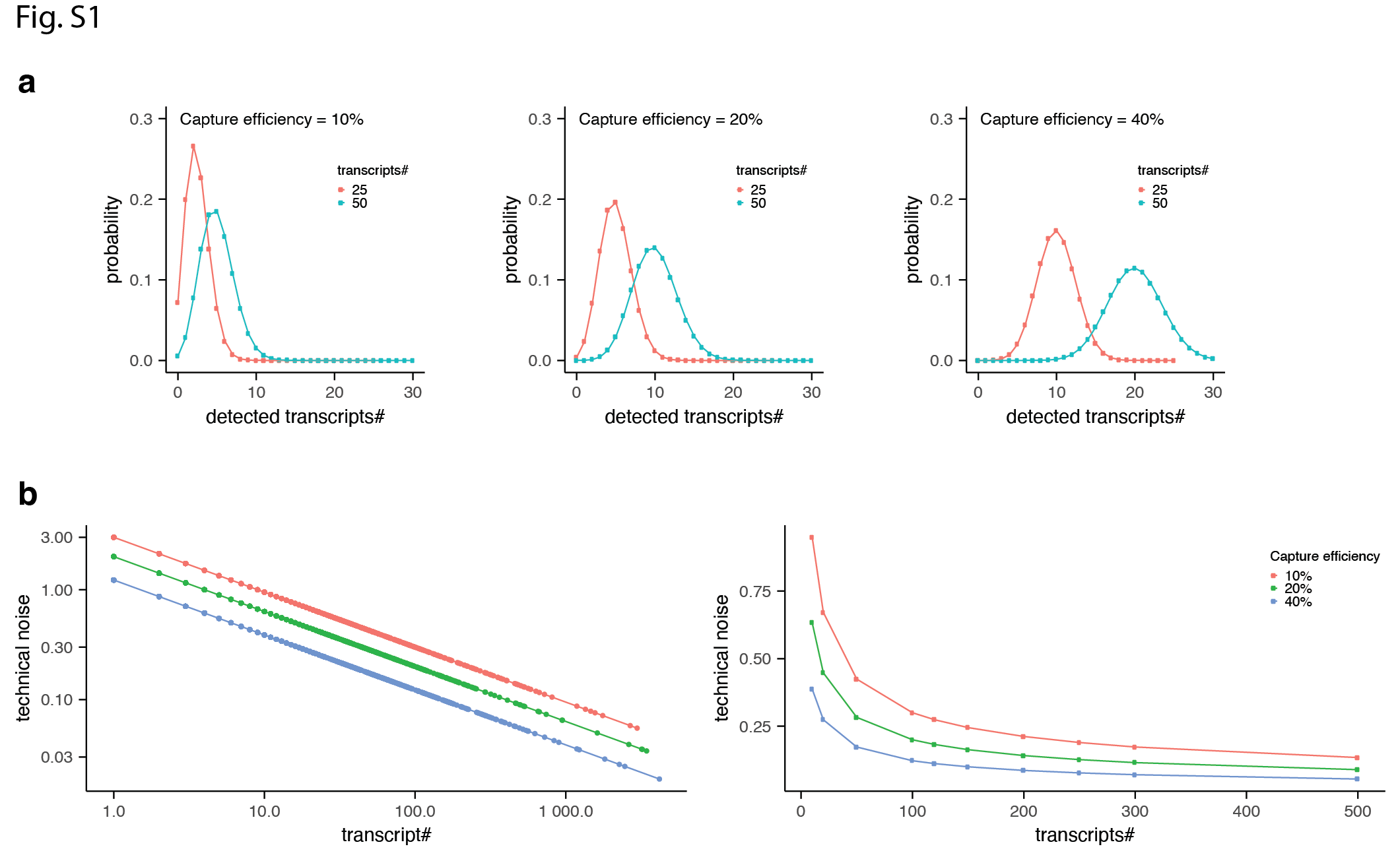


Fig. S1. Simulated performance of scRNA-seq with varying single-molecule capture efficiency. **a**. Single-molecule capture efficiency affect scRNA-seq’s capability to distinguish different RNA levels. **b**. Single-molecule capture efficiency affect the level of technical noise in scRNA-seq data, plotted against single-cell RNA copy numbers in log (left) and linear (right) scales.


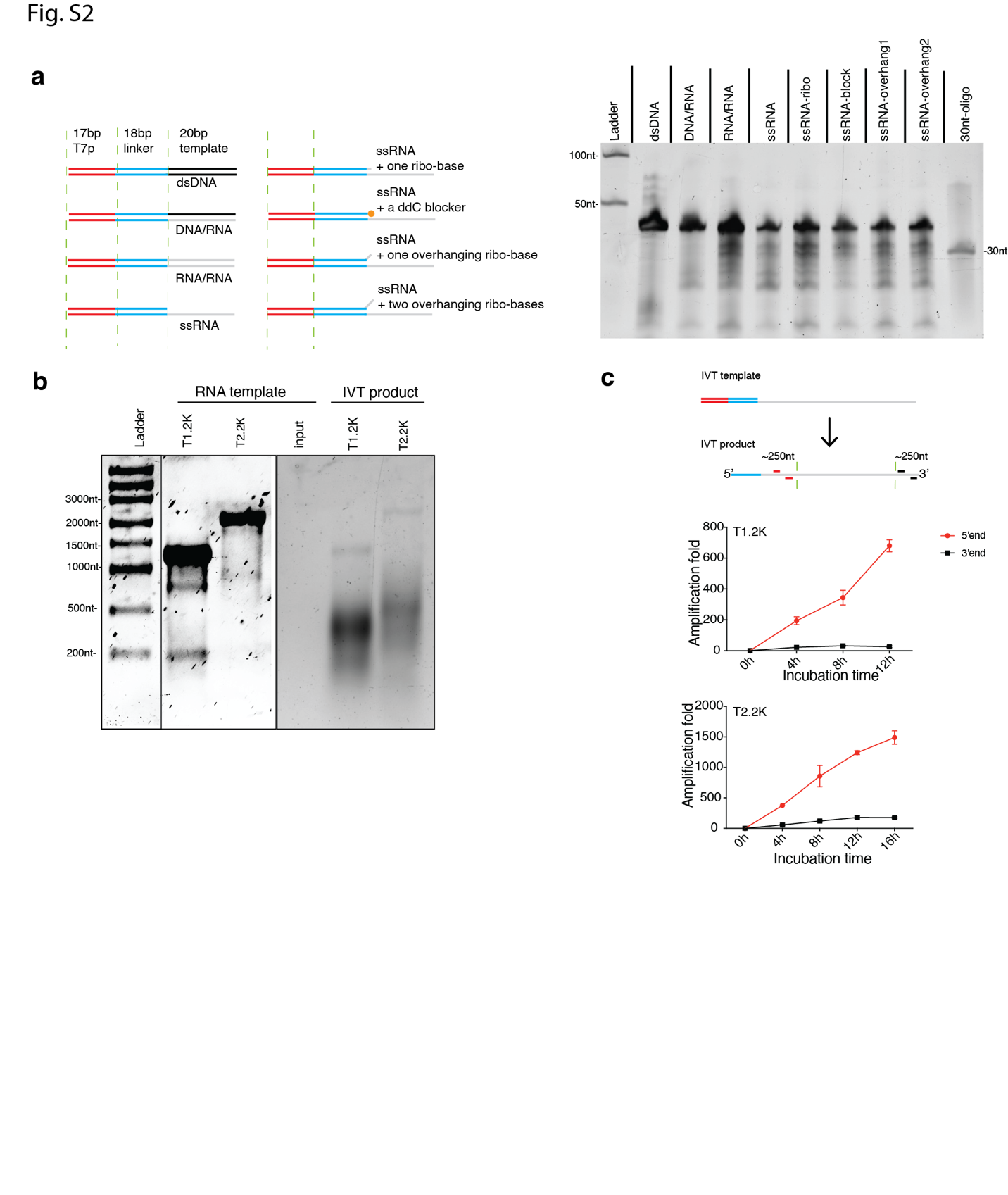


Fig. S2. T7 In vitro transcription (IVT) of ssRNA templates. **a**. T7 IVT products of various short templates (left) resolved by urea polyacrylamide gel (right). **b**. T7 IVT products of long ssRNA templates resolved by denaturing agarose gel. The input lane includes a mixture of two types of T7 IVT templates without any incubation time. **c**. RT-qPCR quantification of T7 IVT products of long ssRNA templates, with the SEM error bar from 3 replicates.


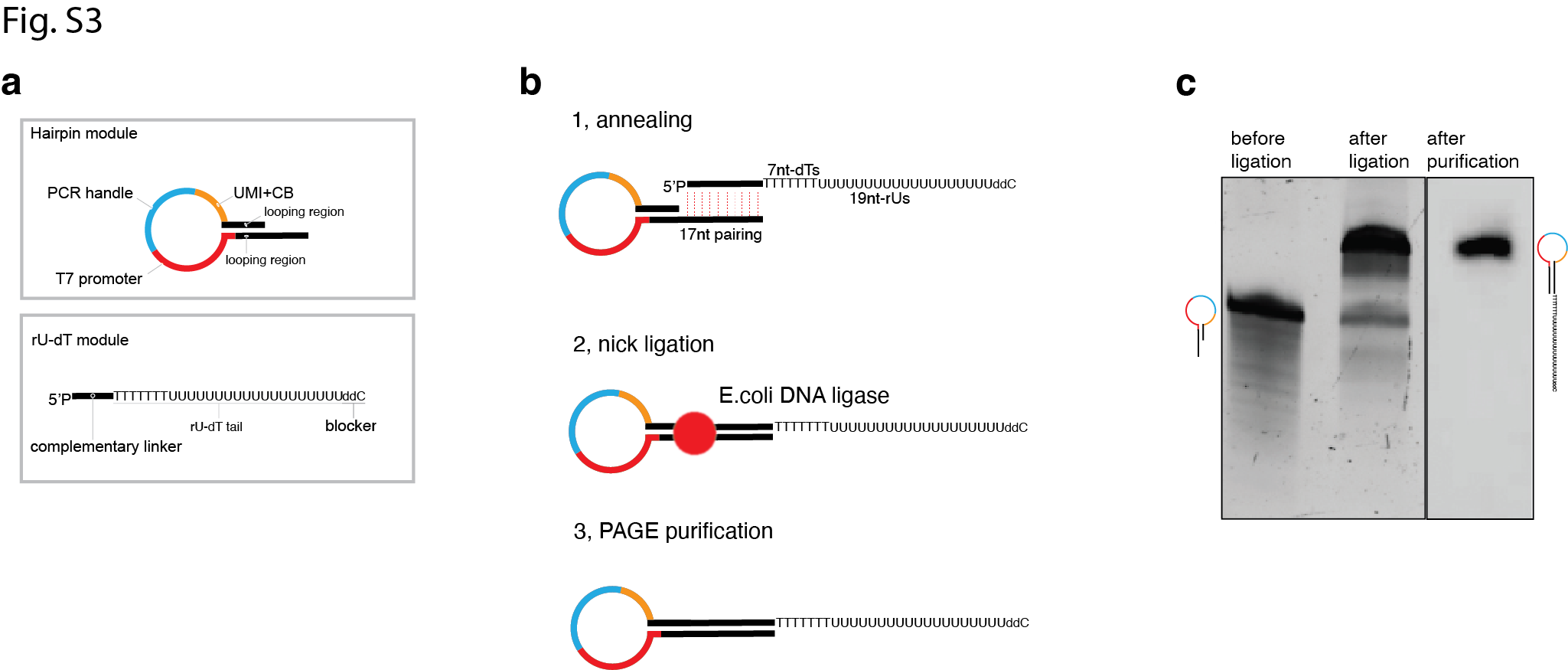


Fig. S3. LAST-seq primer. **a**. Schematic of the LAST-seq primer consisting of a hairpin module (top) and an rU-dT module (bottom). **b**. Making of the LAST-seq primer by annealing and nick ligation between the hairpin module and the rU-dT module, followed by the PAGE purification. **c**. Products of the ligation and PAGE purification.


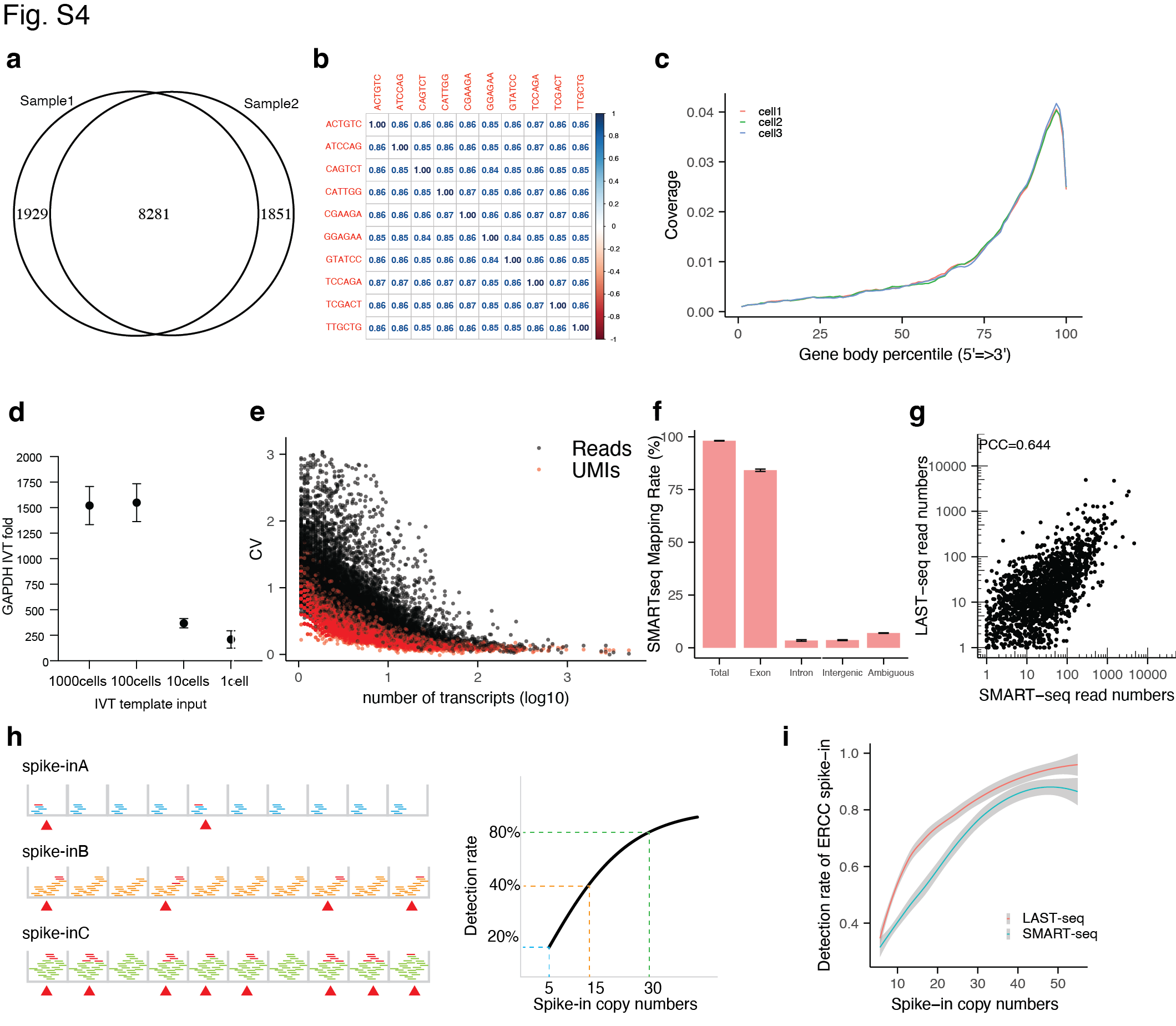


Fig. S4. Performance of LAST-seq. **a**. Number of genes detected in two LAST-seq replicates, each using 15-pg HEK293T-extracted total RNA as the input. **b**. Table of PCC values demonstrating the reproducibility of LAST-seq between 10 replicates, each using 15-pg HEK293T-extracted total RNA as the input. **c**. Sequencing read coverage of the LAST-seq data along the gene body. **d**. RT-qPCR quantification to estimate the IVT amplification fold of GAPDH mRNA molecules, plotted against various input amount for IVT incubation, with the SEM error bar from 3 replicates. **e.** Coefficient of variation (CV)-mean plot to compare the counting by the number of UMIs and by the number of sequencing reads, between 10 replicates. Each dot corresponds to one gene. **f**. Mapping rates of the SMART-seq reads to different genomic regions. The SEM error bar is calculated from 9 cells. **g**. Correlation of RNA level quantified by the number of sequencing reads between LAST-seq and SMART-seq, averaged from 10 and 9 cells, respectively. Each dot corresponds to one gene. **h**. Estimating single-molecule capture efficiency based on the detection rate of each RNA spike-in species, calculated as the fraction of wells (indicated by red triangles) with detected spike-in molecules (labeled in red). **i**. Detection rate comparison between LAST-seq and SMART-seq, with the shadow marking 95% confidential interval from 10 replicates.


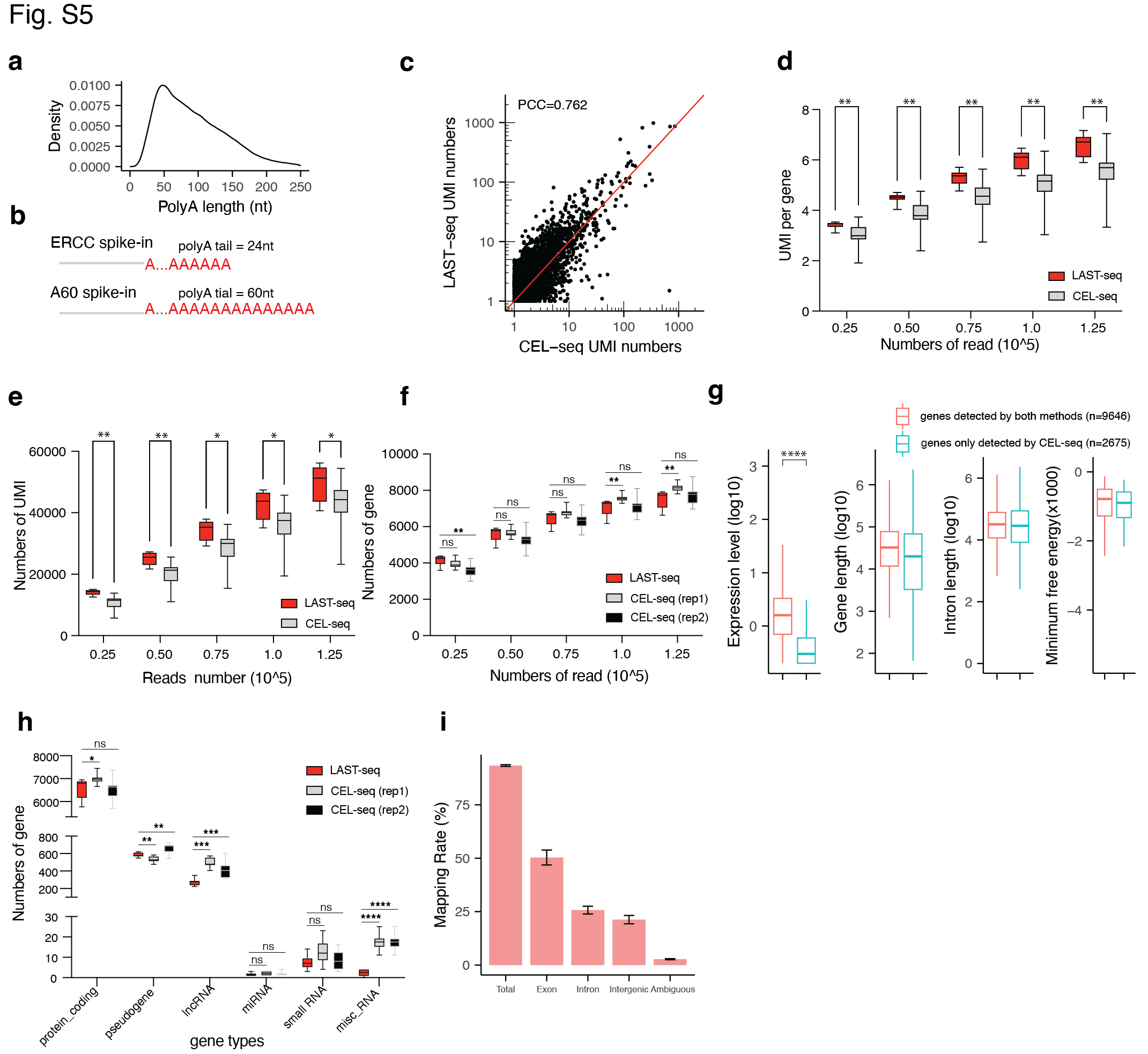


Fig. S5. Comparison between LAST-seq and CEL-seq. **a**. Distribution of mRNA poly-A tail lengths in human cells. **b**. Poly-A tail length of the ERCC and A60 RNA spike-ins. **c**. Correlation of RNA level quantified by UMI numbers between LAST-seq and CEL-seq, both averaged from 10 single cells. Each dot corresponds to one gene. **d**. Number of UMIs per gene comparison between LAST-seq and CEL-seq, both averaged from 10 single cells. **e**. Number of total UMI per cell comparison between LAST-seq and CEL-seq, both averaged from 10 single cells. **f**. Number of detected genes per cell comparison between LAST-seq and CEL-seq, both averaged from 10 single cells. The boxplot shows the median (center line), the 25/75 percentile (bounds), and the minimum/maximum (whiskers). The statistical analysis was performed by the Welch t- test (ns, p > 0.05, *p ≤ 0.05, **p ≤ 0.01, ***p ≤ 0.001, ****p ≤ 0.0001). **g.** Difference in expression level, gene/intron length, and minimum free energy (secondary structure) between genes detected and not detected by LAST-seq. 5% of all genes were randomly selected for secondary structure analysis. The statistical analysis was performed by two-sided Wilcoxon test (****p ≤ 0.0001). **h.** Gene types detected by CEL-seq and LAST-seq. The statistical analysis was performed by Welch t-test (ns, p > 0.05, *p ≤ 0.05, **p ≤ 0.01, ***p ≤ 0.001, ****p ≤ 0.0001). **i**. Mapping rates of the CEL-seq reads to different genomic regions. The SEM error bar is calculated from 10 cells.


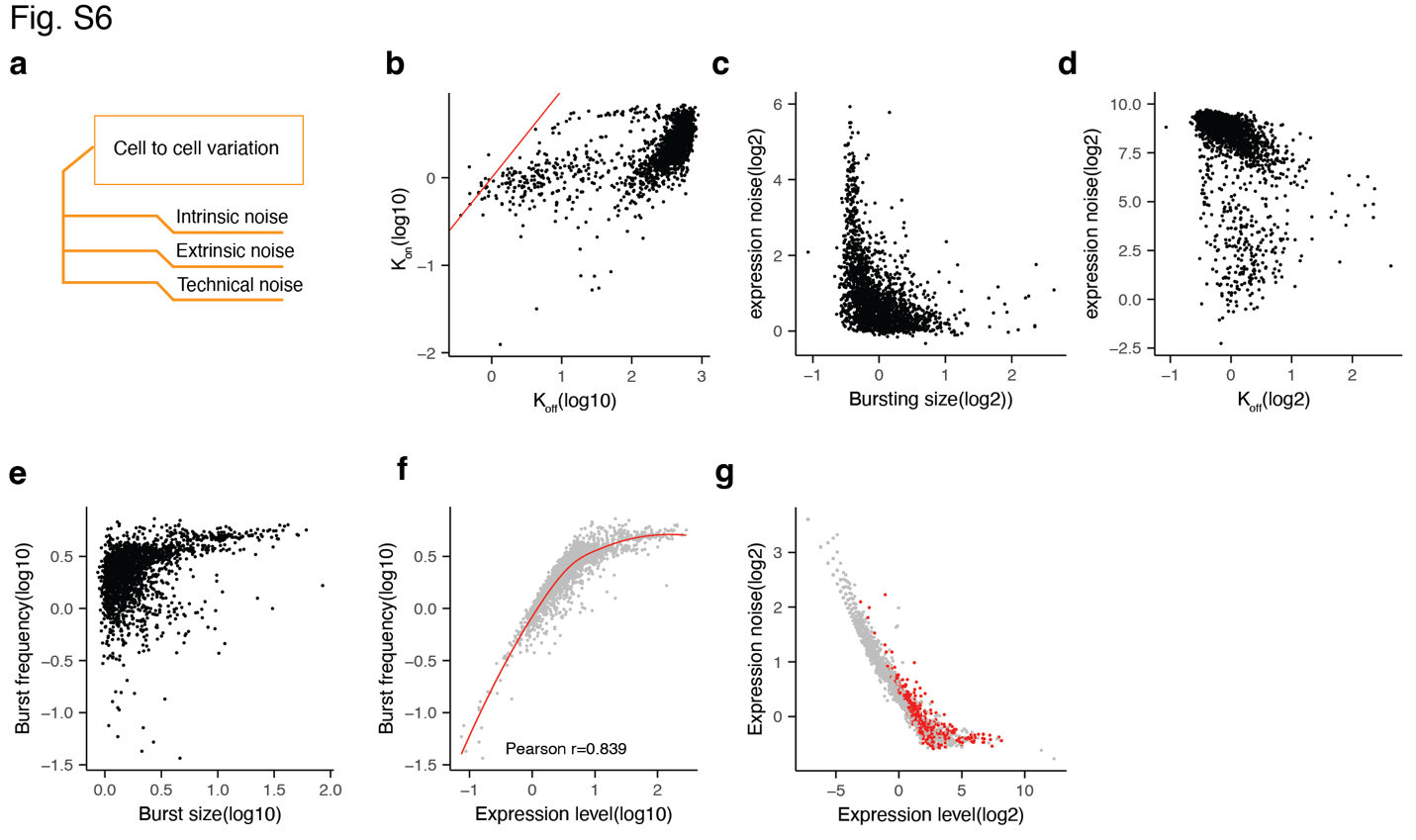


Fig. S6. Transcriptional bursting kinetics in human cells. **a**. Types of noise contributing to the observed cell-to-cell variation. **b**. K_on_/K_off_ plot. The red diagonal line marks where K_on_ equals to K_off_. **c**. Correlation between expression noise and burst size. **d**. Correlation between expression noise and K_off_. **e**. Correlation between burst frequency and burst size. **f**. Correlation between the expression level and burst frequency. **g**. Gene expression noise profile of the haploid human cells plotted against the gene expression level. The red-marked genes passed the curve fitting filter to derive transcriptional burst parameters.


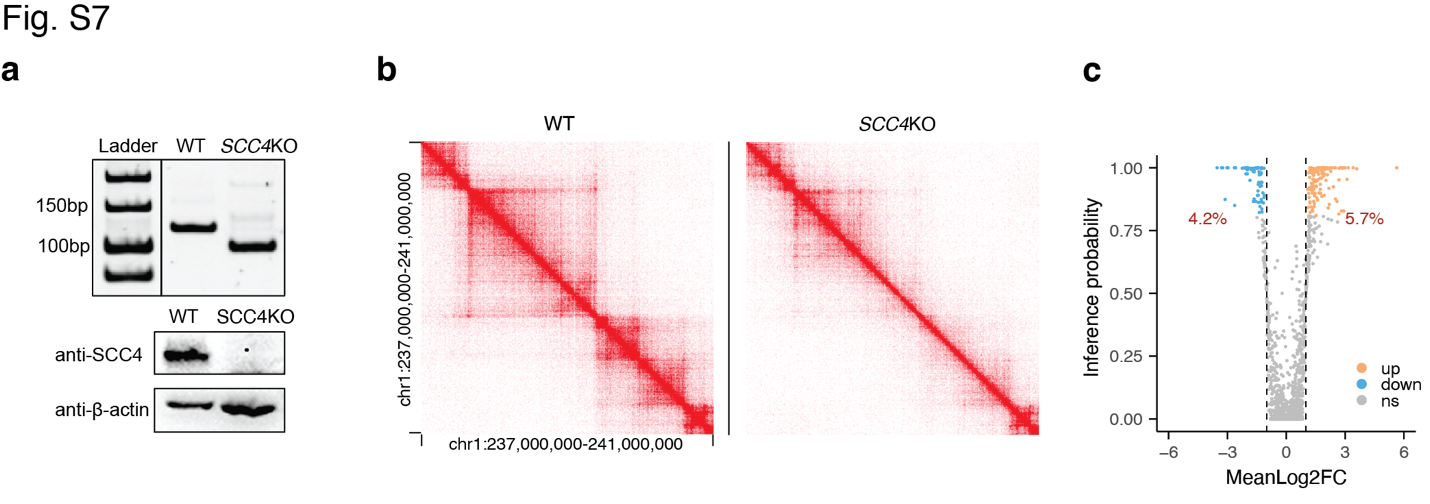


Fig. S7. Chromatin structure and gene expression level in wildtype and *SCC4* knockout haploid human cells. **a**. *SCC4* knockout in the haploid cells validated by genotyping (top) and Western blot (bottom). **b**. TADs disruption demonstrated by the Hi-C contact matrix upon *SCC4* knockout in the haploid cells. **c**. Genes differing in expression levels upon *SCC4* knockout by the Volcano plot.


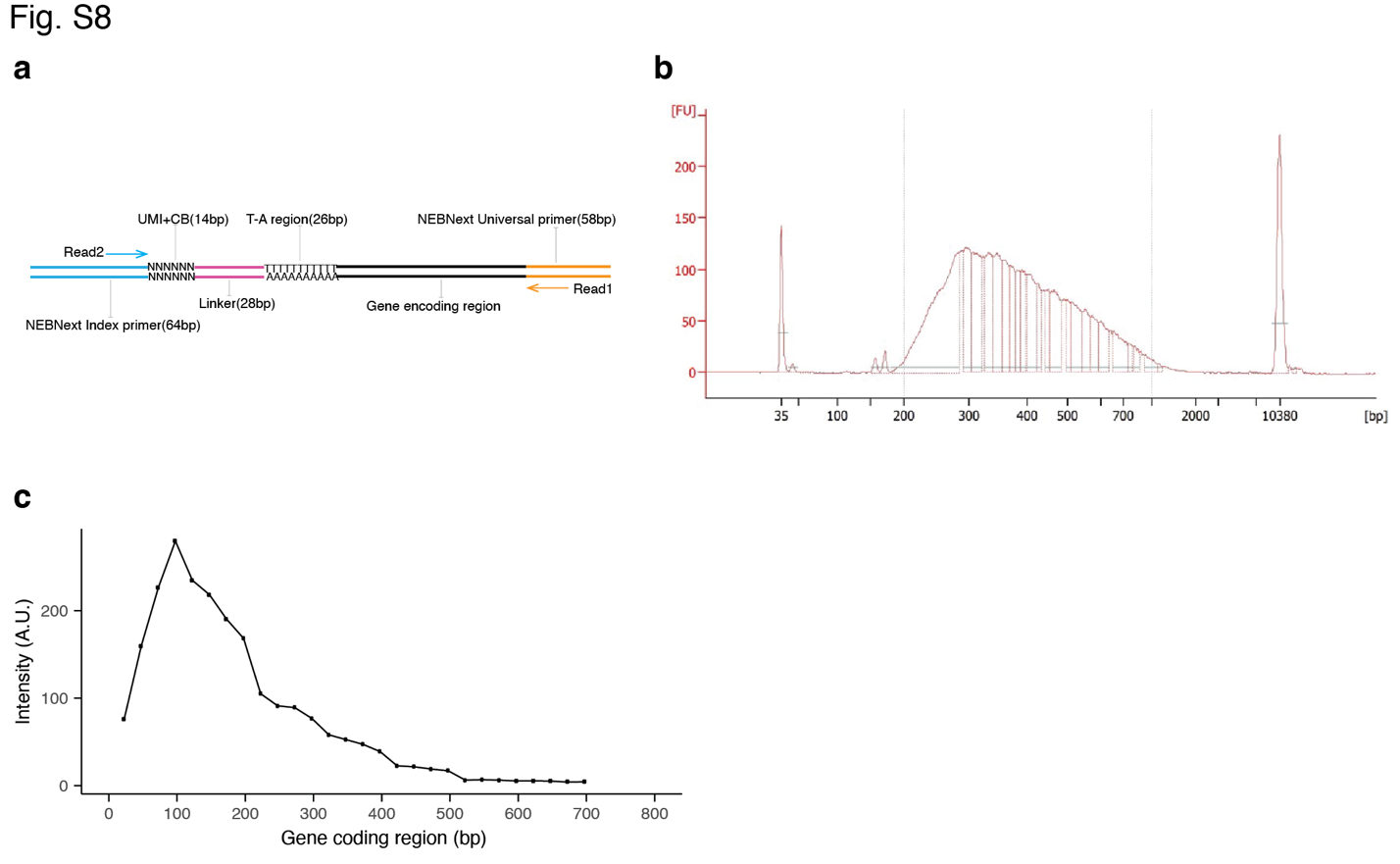


Fig. S8. LAST-seq library. **a**. Components of the DNA fragments in the LAST-seq library upon sequencing. **b**. Typical DNA fragment size distribution of the LAST-seq library. **c**. Typical size distribution of the gene coding region in the LAST-seq library, after subtracting the lengths of Illumina adaptors and other non-coding components.
