# Supplementary material for: LAST-seq: single-cell RNA sequencing by direct amplification of single-stranded RNA without prior reverse transcription and second-strand synthesis
Source: Genome Biol. 2023 Aug 9;24:184. doi: 10.1186/s13059-023-03025-5 (PMC10413806; doi:10.1186/s13059-023-03025-5)
Supplement: Supplementary file 3 — Additional file 3. Review history. [file 13059_2023_3025_MOESM3_ESM.docx]

Review History

**First round of review**

**Reviewer 1**

**Were you able to assess all statistics in the manuscript, including the appropriateness of statistical tests used?** Yes: Authors need to add statistical tests to Fig. 4C/D/F/G

**Were you able to directly test the methods?** No.

**Comments to author:**

LAST-seq review

The authors describe LAST-seq, a new molecular biology workflow for performing scRNA-seq which bypasses mRNA reverse transcription and cDNA second strand synthesis by directly amplifying mRNA molecules using in vitro transcription. The experiments/analyses in the manuscript are as follows:

1. Pilot T7 IVT experiments using different oligo templates which demonstrate applicability of T7 IVT to ssRNA templates — experiments/analyses were well-performed, only minor comments:

* Authors should discuss why the differences in average product lengths (Fig. S2B) and amplification efficiency (Fig. S2C) differs between the T1.2K and T2.2K templates.

* Line 55: "Based on the discovery that T7 IVT can directly amplify ssRNA templates" — Way this is written suggests that the authors discovered this, but amplification of ssRNA via T7 IVT was described by Arnaud-Barbe et al (unless I am under appreciating the novelty of the experiments shown in Fig. S2)

2. SMART-seqV4 comparison experiments which demonstrate LAST-seq technical reproducibility and increased LAST-seq gene detection and ability to capture low-abundance spike-ins relative to SMART-seqV4 — experiments/analyses were well-performed, only minor comments.

* Fig. 1B: Change x-axis label from 'ambiguity' to 'ambiguous' and add axis title. Also consider adding data for other methods to enable reader to compare assess any differences.

* Fig. 1C/D/E/F/G: Consider adding figure titles to facilitate easier interpretation by the reader.

* Fig. S4F (and other figure panels): A bit confused… Does each dot correspond to a different transcript? Perhaps edit the legend text to clarify.

3. CEL-seq2 comparison experiments which demonstrate improved UMIs and similar gene detection efficiency — experiments/analyses were well-performed; one major comment and other minor comments:

* Major comment:

* Fig. S5F does not conclusively demonstrate that "CEL-seq2 is more likely to amplify other types of cellular RNA in addition to the mature mRNA." What types of RNA are the authors referring to? It is also unclear why referencing La Manno et al is relevant here since they do not discuss CEL-seq2 capture of non-mature mRNAs. I suggest that the authors develop this section better — perhaps including comparative analyses of which genes differ between LAST-seq and CEL-seq2 (i.e., differences in gene length? Number of introns? Predicted secondary structure? Etc.)

* Minor Comments:

* Consider moving Fig. 2A/B to SI and replace with experimental schematic to improve figure interpretability.

* Authors could consider discussing (in results or discussion sections) why LAST-seq nUMIs were higher than CEL-seq2 on the A60 spike-ins but not ERCC.

* Red/grey legend denoting LAST-seq/CEL-seq2 should be moved to Fig. 2F/G since those colors are not used in Fig. 2E.

4. eHAP experiments to quantify transcriptional bursting parameters and assess how bursting parameters are influenced by topologically associated domains - experiments/analyses are well-performed, although section needs significant development to improve interpretability; three major comments and one minor comments:

* Major comments:

* Since most readers of this manuscript are unlikely to be experts in gene expression noise and 3D genome organization (like myself), the authors need to do a much better job of introducing a lot of the concepts discussed in this section. For example, the authors should better introduce what the different sources of expression noise are, what TADs are, what compartments A and B are, etc. Also, the authors should explain better why LAST-seq is particularly well-suited to measure bursting parameters and test their TAD hypothesis. Overall, this section felt like a very sharp deviation from the prior sections and was difficult to follow as a reader who is admittedly not well-versed in the gene expression noise / 3D genome organization literature.

* Since scRNA-seq-based analysis of gene expression noise has been performed previously using SMART-seq (Larsson et al), and since one of the promises of LAST-seq is that the method is better able to capture gene expression noise due to decreased technical noise, the authors should at least comment on whether the analyses presented here are an improvement over previous work.

* Authors cannot claim that "no major changes in the gene expression level were detected upon cohesion loss and TAD disruption" when the expression level of 10% of detected genes change (Fig. S7C). Perhaps use GSEA to assess whether the DEGs are functionally-linked or repeat the DEG analysis on random sets of WT and SCC4 KO cells to show that similar numbers of DEGs are detected upon permutation.

* Minor comments:

* To improve interpretation, authors may consider removing genes with <10 UMIs from Fig. 3H and Fig. S6F since lowly expressed genes appear to have good correlation with both burst size and frequency.

* Fig. 4B: Change "low variabilit" to "low variability"

* Fig. 4C/D/F/G: Include statistical tests.

On balance, I believe that LAST-seq represents a sufficiently novel molecular biology workflow for performing scRNA-seq to warrant publication in Genome Biology. The benchmarking experiments comparing LAST-seq to SMART-seq and CEL-seq were well-executed and convincing. I applaud the authors for then using LAST-seq scRNA-seq data to address questions related to gene expression noise that are not usually addressed using single-cell genomics due to high levels of technical noise. However, in my opinion, the way that their analyses are presented - e.g., introducing the concepts, comparing to prior work, etc. - should be better developed prior to publication. Thus, I believe that this manuscript should be conditionally-accepted for publication in Genome Biology assuming that the major comments are adequately addressed in the revised manuscript.

**Reviewer 2**

**Were you able to assess all statistics in the manuscript, including the appropriateness of statistical tests used?** Yes: No problem.

**Were you able to directly test the methods?** No.

**Comments to author:**

In this paper, the authors investigated the influence of reverse transcription (RT) and second-strand synthesis (SSS) efficiency on the performance of in vitro transcription (IVT)-based single-cell RNA sequencing (scRNA-seq) by circumventing the RT/SSS steps. A reasonable interpretation of the findings presented herein is that CEL-seq2 and LAST-seq exhibit nearly identical performance, indicating that the impact of RT/SSS efficiency is negligible. The authors conducted a comparison between Smart-Seq V4 and LAST-seq, focusing on the differences between IVT and template switching combined with PCR, rather than explicitly examining the consequences of omitting the RT/SSS steps.

Mejor concerns:

While the omission of RT/SSS did not substantially enhance performance, the undertaking itself was crucial for discerning which fundamental steps of the scRNA-seq process were critical for optimal results. Consequently, this information will be of widespread interest to both developers and users of scRNA-seq methodologies. Additionally, the latter portion of the paper demonstrates that LAST-seq addresses quantitative issues such as transcriptional bursts, thereby validating the applicability of LAST-seq to real-world challenges, akin to conventional scRNA-seq approaches.

For these reasons, although the results of this paper may not be overwhelmingly positive, it is a suitable study for acceptance and publication in this journal. Nevertheless, we do have some significant concerns regarding the following aspects:

Could you elaborate on why LAST-seq performs comparably to CEL-seq2 after the omission of RT/SSS? For instance, does the efficiency of the novel reaction step counterbalance the effects of excluding RT/SSS? Factors contributing to the efficiency of the new reaction step include ligation efficiency, nick insertion efficiency by RNase H, and extension efficiency by DNA polymerase.

In the comparison between CEL-seq2 and LAST-seq, the latter appears to be inferior with respect to the number of detected genes. Conversely, the unique molecular identifier (UMI) per gene analysis demonstrates LAST-seq's superiority. To reconcile this discrepancy, the authors present Table 1, which reveals that CEL-seq2 sequences genomic DNA. This data constitutes a crucial experiment that bolsters the validity of the authors' argument. It is recommended that the authors provide a more detailed description of the Table 1 experiment in both the main text and the Methods section. Furthermore, it is important to note that these are not the only results obtained by the authors. A similar phenomenon can be observed in Figure 1 of Mereu et al. (2020), where intron reads are unjustifiably inflated. LAST-seq, like CEL-seq2, does not show genomic DNA contamination and over-mapping to Intron, despite the use of IVT. The fact that LAST-seq does not include these suspicious steps is an appealing point.

The spike-in experiments might be unrealistic (Fig. 1d, e, g, i). Typical mammalian cells contain approximately 100,000 copies of RNA molecules, with even the most highly expressed genes having several thousand copies. However, the maximum value on the axis in the UMI experiment figure is 10,000. This figure seems to represent an experiment using an excessive number of RNA molecules. Does this not imply that the quantity of RNA used exceeds the equivalent of a single cell? If so, this experiment would be unsuitable for evaluating the quantitative nature of scRNA-seq and would only be appropriate for examining low-quantity RNA-seq. In such a case, the correlation coefficient would appear artificially high. The authors should clarify the total number of spike-in RNA molecules included in each experiment.

Regarding the performance comparison between LAST-seq and Smart-Seq V4, the superiority of LAST-seq has not been definitively established, as there is no data from Smart-Seq's spike-in experiments conducted under the same experimental conditions as those of LAST-seq.

It is customary to generate a CV-mean plot (as shown in Fig. 2D of Hashimshony et al., 2016) to compare UMI and raw counts.

Minor concern:

The term "CEL-seq2" should be avoided in favor of "CEL-seq."

**Authors Response**

**Point-by-point responses to the reviewers’ comments:**

Reviewer reports:

Reviewer #1: LAST-seq review

*The authors describe LAST-seq, a new molecular biology workflow for performing scRNA-seq which bypasses mRNA reverse transcription and cDNA second strand synthesis by directly amplifying mRNA molecules using in vitro transcription. The experiments/analyses in the manuscript are as follows:

1.      Pilot T7 IVT experiments using different oligo templates which demonstrate applicability of T7 IVT to ssRNA templates — experiments/analyses were well-performed, only minor comments:*

**       Authors should discuss why the differences in average product lengths (Fig. S2B) and amplification efficiency (Fig. S2C) differs between the T1.2K and T2.2K templates.*

**Response:** Thank you for the suggestion. The differences in average product lengths and amplification efficiency were likely resulted from the distinct template sequences, which is also observed in canonical T7 in vitro transcriptional on dsDNA templates, as reported by a previous study (Duftner et al. Genomics. 2008). We have now included some discussion and the reference on this topic in the main text (Line 54).

In addition, we specified the original sequences of T1.2K and T2.2K in the method section (Line 290), providing more details of the sequence difference between the T1.2K and T.2.2K templates.

**       Line 55: "Based on the discovery that T7 IVT can directly amplify ssRNA templates" — Way this is written suggests that the authors discovered this, but amplification of ssRNA via T7 IVT was described by Arnaud-Barbe et al (unless I am under appreciating the novelty of the experiments shown in Fig. S2)*

**Response:** Thank you for pointing out this ambiguity. Yes, Arnaud-Barbe et al described the IVT on short ssRNA templates in the first place. Our study expanded their discovery to much longer ssRNA templates (>20-nt), quantified the linear amplification fold along the time course, and measured the aRNA length to be reaching 200-500 nt, thus enabling its use in direct RNA amplification. We have changed this particular sentence in the main text to distinguish our observation from what was known before our work (Line 58).

2.      SMART-seqV4 comparison experiments which demonstrate LAST-seq technical reproducibility and increased LAST-seq gene detection and ability to capture low-abundance spike-ins relative to SMART-seqV4 — experiments/analyses were well-performed, only minor comments.

**       Fig. 1B: Change x-axis label from 'ambiguity' to 'ambiguous' and add axis title. Also consider adding data for other methods to enable reader to compare assess any differences*.

**Response:** Thank you for the suggestion. We have changed the x-axis label from 'ambiguity' to 'ambiguous' and added “Genomic features” as the x-axis title. We have made the changes in Fig. 1B, as well as in Fig. S4F and Fig. S5I. The mapping rate data of SMART-seq and CEL-seq2 have been included in Fig. S4F and Fig. S5I, respectively.

**       Fig. 1C/D/E/F/G: Consider adding figure titles to facilitate easier interpretation by the reader.*

**Response:** Thank you for the suggestion. We have modified Fig. 1 to add titles, including “technical reproducibility” in Fig. 1C, “uniform amplification of RNA spike-in” in Fig. 1D, “uniform amplification of transcriptome” in Fig. 1E, “counting accuracy by read #” in Fig. 1F, and “counting accuracy by UMI #” in Fig. 1G.

**       Fig. S4F (and other figure panels): A bit confused… Does each dot correspond to a different transcript? Perhaps edit the legend text to clarify.* 

**Response:** We are sorry for the confusion. Yes, each dot corresponds to one gene/transcript in Fig. S4F (now Fig. S4G in the revision). We have added a sentence in the figure legend to clarify. The same change was made to the figure legends of Fig. S5C and Fig. 2D.

3.      CEL-seq2 comparison experiments which demonstrate improved UMIs and similar gene detection efficiency — experiments/analyses were well-performed; one major comment and other minor comments:

*       Major comment:
**       Fig. S5F does not conclusively demonstrate that "CEL-seq2 is more likely to amplify other types of cellular RNA in addition to the mature mRNA." What types of RNA are the authors referring to? It is also unclear why referencing La Manno et al is relevant here since they do not discuss CEL-seq2 capture of non-mature mRNAs. I suggest that the authors develop this section better — perhaps including comparative analyses of which genes differ between LAST-seq and CEL-seq2 (i.e., differences in gene length? Number of introns? Predicted secondary structure? Etc.)*

Response: Thank you for the insight and suggestion. The reference showed that CEL-seq2 will amplify more intronic and intergenic regions than LAST-seq, that is likely due to the amplification of pre-mRNA and noncoding RNA, and even possibly from the nonspecific amplification of genomic DNA, as our data in Table S1 demonstrated. We have now expanded this part in the main text to further elaborate this point (Line 125), according to your suggestion and the other reviewer’s recommendation.

Following your suggestion, we have performed comparative analyses of the detected genes by LAST-seq and CEL-seq2. Among the genes detected by CEL-seq2 but not by LAST-seq, we found they tend to have a lower expression level, but no statistical difference can be claimed in terms of the gene length, intron length, or the stability of the secondary structure (see below Fig. a, and have now included in Fig. S5G). For the stability of the secondary structure, we used the minimum free energy (delta G) to predict the difficulty of forming a stable secondary structure of each RNA transcript (Line 388). Also, we found CEL-seq2 constantly detected more lncRNAs and miscRNAs than LAST-seq (see below Fig. b, and have now included in Fig. S5H), in agreement with the elevated level of intronic and intergenic reads from RNA types other than the mature mRNA in the CEL-seq2 data.

We conclude that LAST-seq may have difficulty in amplifying some lowly expressed genes or certain RNA transcript with a special feature, due to compromised IVT of certain ssRNA templates. On the other hand, CEL-seq2 may suffer from nonspecific amplification of genomic DNA and non-mature RNA, leading to its apparent detection of more genes.

*       Minor Comments:
**       Consider moving Fig. 2A/B to SI and replace with experimental schematic to improve figure interpretability.*

**Response:** Thank you for this great suggestion. We moved the original Fig. 2A/B to Fig. S5A/B.

We then added a new Fig. 2A to demonstrate our experimental schematic, designed to compare the performance of LAST-seq and CEL-seq2 in detecting RNA spike-in species and transcriptomic mRNA molecules.

**       Authors could consider discussing (in results or discussion sections) why LAST-seq nUMIs were higher than CEL-seq2 on the A60 spike-ins but not ERCC.*

**Response:** Thank you for the suggestion. We think the better performance of LAST-seq than CEL-seq2 on A60 spike-ins is due to the enhanced capture efficiency of LAST-seq on RNAs with a longer poly-A tail. In addition to increasing the chance of annealing to the poly-A tail, a longer poly-A tail ensures a stable rA-dT hybrid, which facilitates RNase H nicking at the hybrid region for T7 promoter attachment. Moreover, this observation is also consistent with the better performance of LAST-seq on real transcriptomic mRNA molecules which contains an average of 60 nt poly-A tail, similar to the A60 RNA spike-ins. We have included the relevant discussion in the main text (Line 110).

**       Red/grey legend denoting LAST-seq/CEL-seq2 should be moved to Fig. 2F/G since those colors are not used in Fig. 2E.*

**Response:** Thank you for pointing out this mistake. We have moved the legend to the bottom right of Fig. 2F/G (now Fig. 2E/F in the revision).

4.      eHAP experiments to quantify transcriptional bursting parameters and assess how bursting parameters are influenced by topologically associated domains - experiments/analyses are well-performed, although section needs significant development to improve interpretability; three major comments and one minor comments:

*       Major comments:
**       Since most readers of this manuscript are unlikely to be experts in gene expression noise and 3D genome organization (like myself), the authors need to do a much better job of introducing a lot of the concepts discussed in this section. For example, the authors should better introduce what the different sources of expression noise are, what TADs are, what compartments A and B are, etc. Also, the authors should explain better why LAST-seq is particularly well-suited to measure bursting parameters and test their TAD hypothesis. Overall, this section felt like a very sharp deviation from the prior sections and was difficult to follow as a reader who is admittedly not well-versed in the gene expression noise / 3D genome organization literature.*

**Response:** Thank you for the suggestion. We have re-written multiple parts extensively in the main text to introduce the relevant concepts in more detail, citing a few more references as well (Line 141-151, Line 165, 172). We hope the revised version will be easier to read and to follow for readers not very familiar with 3D chromatin organization and gene expression noise. LAST-seq is a well-suited tool to study transcriptional bursting in the context of 3D chromatin organization, thanks to its accuracy in quantitative characterization of gene expression level in individual cells, and gene expression noise derived from cell-to-cell variation.

**       Since scRNA-seq-based analysis of gene expression noise has been performed previously using SMART-seq (Larsson et al), and since one of the promises of LAST-seq is that the method is better able to capture gene expression noise due to decreased technical noise, the authors should at least comment on whether the analyses presented here are an improvement over previous work.*

**Response:** Thank you for the suggestion. We have now included our comments that LAST-seq enhances the accuracy of probing gene expression noise in the main text (Line 135). If everything else is the same, just using LAST-seq to replace SMART-seq, it will offer some improvement in the analyses by reducing technical noise. However, in reality, the analyses of gene expression noise and transcriptional bursting could be influenced by a variety of factors, many of which also vary between our work and the previous work, such as the extrinsic noise, cell types, the number of cells analyzed, the model, parameters, and algorithm for curve fitting. Also, we studied haploid cells, while previous work studied diploid cells and rely on SNPs to tell apart the transcription of two alleles. As a result, we feel it hard to directly compare our analyses in this work to the analyses in previous work and claim any scientific superiority.

**       Authors cannot claim that "no major changes in the gene expression level were detected upon cohesion loss and TAD disruption" when the expression level of 10% of detected genes change (Fig. S7C). Perhaps use GSEA to assess whether the DEGs are functionally-linked or repeat the DEG analysis on random sets of WT and SCC4 KO cells to show that similar numbers of DEGs are detected upon permutation.*

**Response:** Thank you for pointing out this confusion and offering a solution. The ~10% of genes as DEGs are real, as we further tested the permutation dataset to show they are gone after permutation. This is consistent with previous reports upon SCC4 depletion in other cell types, where people consider such impact on the expression level of ~10% of genes to be “limited” instead of “major”. To avoid the confusion by the inappropriate claim of “no major changes” with a subjective definition, we now changed the sentence in the main text to explicitly say that ~10% of genes changed their expression level as DEGs (Line 194).

Moreover, according to your suggestion, we performed GO analysis on these DEGs to explore their functional relevance. We found no significant enrichment (q-value < 0.1) of GO items in biological process (BP) and molecular function (MF) categories and only 5 GO items including tens of genes got enriched in the cellular component (CC) category, indicating these DEGs were largely functionally irrelevant.

*       Minor comments:
**       To improve interpretation, authors may consider removing genes with <10 UMIs from Fig. 3H and Fig. S6F since lowly expressed genes appear to have good correlation with both burst size and frequency.*

**Response:** Thank you for the suggestion. We further edited the main text (Line 162) to clarify the interpretation, especially separating lowly expressed genes and highly expressed genes, since the two categories of genes have a different behavior. We still keep all genes in the figures in order to offer the readers a comprehensive picture.

**       Fig. 4B: Change "low variabilit" to "low variability"*

**Response:** Thank you for pointing out this typo. We now have it corrected.

**       Fig. 4C/D/F/G: Include statistical tests.*

**Response:** Thank you for the suggestion. We have included the statistical tests in Fig. 4C/D/F/G, and explained them in the corresponding figure legend.

On balance, I believe that LAST-seq represents a sufficiently novel molecular biology workflow for performing scRNA-seq to warrant publication in Genome Biology. The benchmarking experiments comparing LAST-seq to SMART-seq and CEL-seq were well-executed and convincing. I applaud the authors for then using LAST-seq scRNA-seq data to address questions related to gene expression noise that are not usually addressed using single-cell genomics due to high levels of technical noise. However, in my opinion, the way that their analyses are presented - e.g., introducing the concepts, comparing to prior work, etc. - should be better developed prior to publication. Thus, I believe that this manuscript should be conditionally-accepted for publication in Genome Biology assuming that the major comments are adequately addressed in the revised manuscript.

We thank the reviewer for all the comments and valuable suggestions. We hope this revised version according to your advice has improved our presentation, and made the manuscript easier to follow by future readers.

Reviewer #2: In this paper, the authors investigated the influence of reverse transcription (RT) and second-strand synthesis (SSS) efficiency on the performance of in vitro transcription (IVT)-based single-cell RNA sequencing (scRNA-seq) by circumventing the RT/SSS steps. A reasonable interpretation of the findings presented herein is that CEL-seq2 and LAST-seq exhibit nearly identical performance, indicating that the impact of RT/SSS efficiency is negligible. The authors conducted a comparison between Smart-Seq V4 and LAST-seq, focusing on the differences between IVT and template switching combined with PCR, rather than explicitly examining the consequences of omitting the RT/SSS steps.

Major concerns:
While the omission of RT/SSS did not substantially enhance performance, the undertaking itself was crucial for discerning which fundamental steps of the scRNA-seq process were critical for optimal results. Consequently, this information will be of widespread interest to both developers and users of scRNA-seq methodologies. Additionally, the latter portion of the paper demonstrates that LAST-seq addresses quantitative issues such as transcriptional bursts, thereby validating the applicability of LAST-seq to real-world challenges, akin to conventional scRNA-seq approaches.
For these reasons, although the results of this paper may not be overwhelmingly positive, it is a suitable study for acceptance and publication in this journal. Nevertheless, we do have some significant concerns regarding the following aspects:

We thank the reviewer for the insight and evaluation.

*Could you elaborate on why LAST-seq performs comparably to CEL-seq2 after the omission of RT/SSS? For instance, does the efficiency of the novel reaction step counterbalance the effects of excluding RT/SSS? Factors contributing to the efficiency of the new reaction step include ligation efficiency, nick insertion efficiency by RNase H, and extension efficiency by DNA polymerase.*

**Response:** Thank you for raising this point. We analyzed a few potential reasons that may compromise the performance of LAST-seq, thus counterbalancing the gains of excluding RT/SSS upon its comparison to CEL-seq2.

(1) The efficiency of T7 promoter attachment. Specifically, the annealing efficiency, the nicking efficiency by RNase H, and the extension efficiency by DNA polymerase. We found the overall T7 promoter attachment efficiency is 60-70% (data see below). Since the RT efficiency of CEL-seq2 using SuperScriptII is 25%-50% (Zucha et al. Clinical Chemistry.2020), plus some more loss in the SSS step, the overall efficiency of CEL-seq2’s RT/SSS would be less than 25%-50%. As a result, replacing RT/SSS with T7 promoter attached by nicking/extension is beneficial.

To measure the attachment efficiency of T7 promoter, we assembled the LAST-seq primer and FAM-labeled RNA template at the molar ratio of 1.5:1, followed by nicking and extension reaction. The original RNA template would be nicked and elongated to ~150 nt after T7 promoter attachment. The FAM intensity is linearly and positively correlated to the number of molecule and thus could be used to estimate the percentage of molecules with T7 promoter attached.

(2) The integrity of the RNA template before IVT. Specifically, in LAST-seq, the RNA template could be partially degraded before IVT, due to environmental exposure upon vacuum-evaporation and RNA purification. This issue is less of a concern in CEL-seq2, where RNA was converted to cDNA by RTase at the very beginning, and cDNA is more stable than RNA. We reasoned that performing LAST-seq in an RNase-free and ultra-clean environment will be beneficial, but such conditions may not be easily or always available in a regular biology lab setting.

(3) The efficiency of IVT linear amplification. As shown by Conrad et al (communications biology, 2020), increasing IVT linear amplification efficiency by optimizing the T7 promoter could improve the performance of CEL-seq2, indicating that the compromised IVT linear amplification fold on ssRNA templates may negatively influence the performance of LAST-seq. Further optimization of the T7 IVT efficiency on ssRNA templates, including screening for the most efficient IVT conditions and T7 RNAP enzyme engineering, will be beneficial for LAST-seq.

Overall, we think the gain by omitting RT/SSS in LAST-seq was likely counterbalanced by RNA template degradation before IVT and the relatively low IVT amplification efficiency, leading to a quite marginal improvement in the performance of LAST-seq when compared to CEL-seq2, as we performed the LAST-seq assay using the regular T7 IVT reaction and in a regular biology lab setting.

*In the comparison between CEL-seq2 and LAST-seq, the latter appears to be inferior with respect to the number of detected genes. Conversely, the unique molecular identifier (UMI) per gene analysis demonstrates LAST-seq's superiority. To reconcile this discrepancy, the authors present Table 1, which reveals that CEL-seq2 sequences genomic DNA. This data constitutes a crucial experiment that bolsters the validity of the authors' argument. It is recommended that the authors provide a more detailed description of the Table 1 experiment in both the main text and the Methods section. Furthermore, it is important to note that these are not the only results obtained by the authors. A similar phenomenon can be observed in Figure 1 of Mereu et al. (2020), where intron reads are unjustifiably inflated. LAST-seq, like CEL-seq2, does not show genomic DNA contamination and over-mapping to Intron, despite the use of IVT. The fact that LAST-seq does not include these suspicious steps is an appealing point.*

**Response:** Thank you for the great recommendation. To evaluate the genomic DNA contamination, we performed LAST-seq and CEL-seq2 on both extracted total RNA and genomic DNA of 10-cell equivalent, and estimated the proportion of genomic DNA contamination of the two assays, according to the final library yield. We have now included a detailed description of this effort in the Methods section (Line 383), and a brief explanation in the main text (Line 126). We have also included the reference as the reviewer suggested, together with our own data, to show the potential gDNA contamination issue in CEL-seq2.

We think it possible that, in CEL-seq2, once the ssDNA primers nonspecifically anneal to the genomic DNA, RT can also occur on the DNA template, so that the T7 promoter has a good chance of being attached to the DNA fragments, leading to gDNA amplification. In contrast, once the LAST-seq primer anneal to the genomic DNA, the poly-rU region of the LAST-seq primer would be digested by RNase H, leading to primer dissociation and failure of T7 promoter attachment. As a result, despite using IVT, LAST-seq has a similar performance to SMART-seq in this aspect, while CEL-seq2 potentially have more gDNA contamination. But we also reasoned that, even if such genomic contamination is an issue in CEL-seq, it seems not to influence the interpretation of most scRNA-seq applications.

*The spike-in experiments might be unrealistic (Fig. 1d, e, g, i). Typical mammalian cells contain approximately 100,000 copies of RNA molecules, with even the most highly expressed genes having several thousand copies. However, the maximum value on the axis in the UMI experiment figure is 10,000. This figure seems to represent an experiment using an excessive number of RNA molecules. Does this not imply that the quantity of RNA used exceeds the equivalent of a single cell? If so, this experiment would be unsuitable for evaluating the quantitative nature of scRNA-seq and would only be appropriate for examining low-quantity RNA-seq. In such a case, the correlation coefficient would appear artificially high. The authors should clarify the total number of spike-in RNA molecules included in each experiment.*

**Response:** Thank you for raising this question. As shown by Islam et al (Nat Methods. 2014), a typical mammalian cell contain 100,000 to 1,000,000 copies of mRNA molecules. We thought HEK293T was probably on the right side of the range, and therefore we spiked ~60,000 molecules in total across 1 copy to 18,000 copies to a single cell lysate. Apart from 4 spike-in species (containing 9000 copies and 18,000 copies) which seem to be unrealistic in real genes, the copy numbers of the remaining spike-in species resemble the expression level of real genes in human cells. We have included the detailed information in the Method section (Line 346), describing that we have added “1 µl of 1:1,000,000 diluted ERCC spike-in or 1:20,000 diluted A60 spike-in”.

To further validate, we also found that, without the 4 spike-in species that may be unrealistically high copy number than typical genes found in human cells, the correlation coefficient is still quite high, thus not affecting our conclusion in Fig. 1G.

*Regarding the performance comparison between LAST-seq and Smart-Seq V4, the superiority of LAST-seq has not been definitively established, as there is no data from Smart-Seq's spike-in experiments conducted under the same experimental conditions as those of LAST-seq.*

**Response:** Thank you for raising this question. We performed the spike-in experiment using Smart-seq and compared the detection rate with that of LAST-seq. Due to the lack of UMI information in Smart-seq, we calculated the spike-in detection rate by counting the fraction of wells where each spike-in species can be detected (Fig. S4G, now Fig. S4H in revised version). As expected, LAST-seq has a higher spike-in detection rate than Smart-seq (Fig. S4H, now Fig.S4I in revised version), which is consistent with its better performance in gene detection.

*It is customary to generate a CV-mean plot (as shown in Fig. 2D of Hashimshony et al., 2016) to compare UMI and raw counts.*

**Response:** Thank you for the great suggestion. We have generated a CV-mean plot to compare UMI and raw counts as below. As expected, UMI counting has a smaller CV than that of read counting due to the removal of amplification bias. We now have this figure included in Fig. S4E, and Line 77.

Minor concern:
*The term "CEL-seq2" should be avoided in favor of "CEL-seq."*

**Response:** Thank you for the suggestion. We have now replaced CEL-seq2 with CEL-seq.

**Second round of review**

**Reviewer 1**

The authors did a great job on the revised manuscript and addressed all of my previous comments.

**Reviewer 2**

The authors have courteously responded to the comments, meticulously addressing the majority of concerns raised.

Minor concerns:

The authors effectively illustrate that CEL-seq2 introduces genomic DNA contamination, a problem absent in LAST-seq. Such contamination could potentially inflate the number of genes detected in an unjustified manner.

The likelihood of contaminated genomic DNA being assigned to a gene region is comparatively lower than transcribed RNA-derived reads being attributed to the same.

Therefore, the observed increment in the number of detected genes in CEL-seq2 might be spuriously inflated, mainly due to an undue rise in low-expressed genes.

If the above hypothesis can be convincingly demonstrated, it might elucidate why LAST-seq excels in the number of detectable UMIs yet seemingly lags in the number of detected genes. In essence, it might turn out that LAST-seq isn't actually deficient in gene detection.

For instance, it would be beneficial to demonstrate the extent of overlap between genes detected by Bulk RNA-seq and those identified by CEL-seq2 and LAST-seq respectively. This would provide a comprehensive comparison and shed light on potential disparities in gene detection among these techniques.

In my previous feedback, I mistakenly suggested a unification of notation to CEL-seq. However, I have come to realize that if the authors are implementing the CEL-seq2 protocol in lieu of CEL-seq, it would be more apt to consistently use CEL-seq2 as the standardized notation throughout.

**Authors Response**

**Point-by-point responses to the reviewers’ comments:**

Reviewer #1: The authors did a great job on the revised manuscript and addressed all of my previous comments.

Reviewer #2: The authors have courteously responded to the comments, meticulously addressing the majority of concerns raised.

We deeply appreciate reviewer#1 and reviewer#2 for their valuable comments and suggestions which significantly improve our manuscripts.

Minor concerns:
*The authors effectively illustrate that CEL-seq2 introduces genomic DNA contamination, a problem absent in LAST-seq. Such contamination could potentially inflate the number of genes detected in an unjustified manner.

The likelihood of contaminated genomic DNA being assigned to a gene region is comparatively lower than transcribed RNA-derived reads being attributed to the same.

Therefore, the observed increment in the number of detected genes in CEL-seq2 might be spuriously inflated, mainly due to an undue rise in low-expressed genes.

If the above hypothesis can be convincingly demonstrated, it might elucidate why LAST-seq excels in the number of detectable UMIs yet seemingly lags in the number of detected genes. In essence, it might turn out that LAST-seq isn't actually deficient in gene detection.

For instance, it would be beneficial to demonstrate the extent of overlap between genes detected by Bulk RNA-seq and those identified by CEL-seq2 and LAST-seq respectively. This would provide a comprehensive comparison and shed light on potential disparities in gene detection among these techniques.*

**Response:** Thank you for providing the solutions to explore the potential gDNA contamination issue in CEL-seq. We found three indirect cues supporting the above hypothesis:

(1) Genes exclusively detected by CEL-seq show predominantly low expression levels (Fig S5g).
(2) Performing CEL-seq on purified gDNA resulted in the detection of 7668 genes, with 78.7% overlapping with transcribed genes detected by CEL-seq and 69.4% overlapping with transcribed genes detected by LAST-seq.
(3) Comparing genes detected by LAST-seq and CEL-seq to publicly available bulk RNA-seq data of the same cell line showed that 83.7% of LAST-seq genes overlapped with bulk RNA-seq genes, while 76.9% of CEL-seq genes overlapped with bulk RNA-seq genes.

*In my previous feedback, I mistakenly suggested a unification of notation to CEL-seq. However, I have come to realize that if the authors are implementing the CEL-seq2 protocol in lieu of CEL-seq, it would be more apt to consistently use CEL-seq2 as the standardized notation throughout.*

**Response:** We’ve emphasized the CEL-seq protocol version in both the main text and in the method section while using the notation of CEL-seq to maintain consistency with SMART-seq notation. We thought the previous suggestion was quite reasonable since CEL-seq represents a specific strategy for implementing single-cell RNA-seq while CEL-seq2 represents an advanced protocol.
